# Changing Epidemiology of Refractive Disorders in the Western Pacific Region, 1990–2023: Evidence From the Global Burden of Disease Study 2023

**DOI:** 10.1155/joph/1397428

**Published:** 2026-09-24

**Authors:** Yuqi Jiang, Shinan Wu, Yuling Chen, Jinzhi Peng, Zuguo Liu, Yalin Wu

**Affiliations:** ^1^ Xiamen Eye Center and Eye Institute of Xiamen University, School of Medicine, Xiamen University, Xiamen Fujian, 361003, China, xmu.edu.cn; ^2^ Fujian Provincial Key Laboratory of Ophthalmology and Visual Science, Fujian Engineering and Research Center of Eye Regenerative Medicine, School of Medicine, Eye Institute of Xiamen University, Xiamen University, Xiamen Fujian, 361102, China, xmu.edu.cn; ^3^ Shenzhen Research Institute of Xiamen University, Shenzhen Guangdong, 518057, China, xmu.edu.cn

**Keywords:** disease burden, epidemiology, global burden of disease, population ageing, refractive disorders, Western Pacific Region

## Abstract

**Introduction:**

Refractive disorders (RDs) are a major cause of vision impairment worldwide. Although age‐standardised rates have generally declined, the absolute burden, demographic drivers and future trends of RDs in the Western Pacific Region (WPR) remain incompletely characterised. This study assessed the long‐term epidemiological patterns and projected burden of RDs in the WPR using data from the Global Burden of Disease Study 2023 (GBD 2023).

**Materials and Methods:**

Data on prevalent cases, prevalence and disability‐adjusted life years (DALYs) due to RDs from 1990 to 2023 were obtained from GBD 2023. Age‐standardised prevalence rates (ASPRs) and age‐standardised DALY rates (ASDRs) were evaluated globally and across 31 countries and territories in the WPR. Temporal trends were assessed using estimated annual percentage change and joinpoint regression. Age‐ and sex‐specific patterns were examined, and Shapley decomposition was used to quantify the contributions of population growth, population ageing and epidemiological change. ARIMA models were used to project ASPR and ASDR to 2030.

**Results:**

In the WPR, prevalent cases increased from 23.45 million in 1990 to 39.75 million in 2023, while ASPR declined from 1606.27 to 1382.90 per 100,000 population. DALYs increased from 1.09 million to 1.75 million, whereas ASDR declined from 76.00 to 59.85 per 100,000. Age‐specific burden increased markedly with age, and females generally experienced a higher burden than males, particularly in middle and older age groups. Joinpoint analyses identified long‐term declines in ASPR and ASDR but modest rebounds after 2019. Population growth was the dominant positive contributor to increasing absolute burden in most WPR countries, while population ageing contributed substantially, particularly in East Asia and among females; epidemiological change generally offset part of these demographic increases. By 2030, ASPR and ASDR in the WPR were projected to decline further to 1317.70 (95% PI 1282.64–1352.76) and 55.00 (95% PI 52.66–57.34) per 100,000, respectively.

**Conclusion:**

Despite declining age‐standardised rates, the absolute burden of RDs in the WPR continues to increase under demographic pressure. Continued population growth and ageing, together with persistent age and sex disparities, indicate that declining rates alone may underestimate future eye‐care needs. Monitoring both age‐standardised and absolute burden, alongside effective refractive error coverage, will be important for regional planning and progress towards the WHO 2030 targets.

## 1. Introduction

Refractive disorders (RDs) refer to a group of ocular conditions in which abnormalities of the eye’s refractive system prevent parallel light rays from being accurately focused on the retina, resulting in reduced visual acuity. They encompass myopia, hyperopia, astigmatism, presbyopia and other disorders of refraction and accommodation. The human refractive system is primarily composed of the cornea and the crystalline lens: the cornea provides most of the eye’s fixed refractive power, while the crystalline lens enables dynamic accommodation through changes in its shape [1]. When the refractive power of the eye does not match the axial length, or when structural abnormalities of the cornea or lens are present, RDs can occur. Previous studies have shown that alterations in corneal morphology can markedly impair visual function and are closely associated with RDs [2].

RDs are a major cause of vision impairment worldwide. According to the GBD 2019 analysis of blindness and vision impairment, an estimated 43.3 million people were blind globally and 295 million were living with moderate or severe vision impairment in 2020 [3]. The World Health Organization (WHO) estimates that at least 2.2 billion people worldwide are affected by vision impairment or blindness, of whom nearly 1 billion have vision problems that are either preventable or have not yet been adequately addressed [4, 5]. Epidemiological evidence consistently indicates that RDs are among the two leading causes of moderate and severe vision impairment, and their disease burden has continued to increase over recent decades. In China, uncorrected RDs are the leading cause of vision impairment and the second leading cause of blindness [6].

Myopia is the most common type of RD, and its prevalence has shown a marked upward trend globally. Evidence from the GBD and related studies indicates that the prevalence of RDs and their associated disability‐adjusted life years (DALYs) have continued to increase between 1990 and 2021 [3]. A study published in 2019 highlighted that the disease burden of uncorrected RDs among adolescents was particularly substantial during 1990–2019. It is projected that by 2050, approximately half of the global population will be affected by myopia, with the prevalence of high myopia also expected to rise markedly [7]. Asian countries, especially China, bear the heaviest burden of myopia; previous estimates suggest that around 46% of vision impairment among Chinese children is attributable to RDs [6].

Uncorrected RDs not only impair visual function but also have far‐reaching effects on education, labour force participation and quality of life [8]. Globally, only about 36% of individuals with distance vision impairment due to RDs have access to appropriate spectacle correction, and more than 800 million people are affected by near‐vision impairment caused by presbyopia. It is projected that by 2030, the number of people with presbyopia will reach 2.1 billion worldwide, while cases of myopia are expected to increase to 3.36 billion. The economic consequences of uncorrected RDs are substantial: annual productivity losses attributable to myopia and presbyopia alone are estimated at 244.0 billion dollars and 25.4 billion dollars, respectively, whereas the global financing gap required to meet unmet needs for spectacle correction is estimated to be only about 16.0 billion dollars.

The Western Pacific Region (WPR) is one of the areas with the highest burden of RDs globally [9], particularly myopia. Adolescents in this region experience high prevalence of myopia, which has been associated with social environments, educational pressure and inequalities in access to eye‐care services [7, 10]. To address these challenges, WHO launched the SPECS 2030 initiative in May 2024 to support Member States in improving access to effective refractive error services and achieving the global 2030 target for effective refractive error coverage. However, comprehensive assessments of the prevalence, temporal trends and disease burden of RDs in the WPR remain limited. Therefore, using data from the GBD 2023, this study systematically evaluated the prevalence, temporal trends and disease burden of RDs in the WPR from 1990 to 2023, providing evidence to inform regional eye health policy development and resource allocation.

## 2. Materials and Methods

### 2.1. Overview

The GBD 2023 systematically estimated years lived with disability (YLDs), years of life lost (YLLs) and DALYs for 375 diseases and injuries and assessed the burden attributable to 88 modifiable risk factors. Of the more than 310,000 data sources used in GBD 2023, approximately 120,000 contributed to estimates of disease and injury burden and about 59,000 to risk factor assessments, drawing on data from vital registration systems, population‐based surveys, disease registries and published literature [11]. All data were synthesised using the Disease Modelling Meta‐Regression framework (DisMod‐MR 2.1), in combination with comparative risk assessment methods, to ensure comparability of estimates across locations and time periods [12]. For each estimated metric, 95% uncertainty intervals (UIs) were calculated based on draws from the posterior distribution, reflecting uncertainty arising from data quality, model specification and parameter estimation [13].

### 2.2. Data Sources and Measures

This study used data from the GBD 2023 to conduct a descriptive epidemiological analysis of the burden of RDs at the global level and in the WPR from 1990 to 2023. In GBD 2023, RDs correspond to the cause ‘Refraction disorders’ (Cause ID 999; ICD‐10 H52–H52.7), encompassing myopia, hyperopia, astigmatism, presbyopia and other disorders of refraction and accommodation within this code range. Detailed disease codes and classification criteria are provided in Appendix Table 1. The main indicators analysed included the number of prevalent cases, prevalence and DALYs, reflecting the non‐fatal disease burden attributable to RDs [14].

Age‐standardised prevalence rates (ASPR) and age‐standardised DALY rates (ASDR) were selected as the primary evaluation metrics to remove the influence of differences in population age structure across regions [15]. Age standardisation was performed using the GBD 2023 world standard population, with the corresponding age‐specific standard population weights applied consistently across locations and years. The age‐standardised rates (ASRs) were calculated according to the standard GBD methodology, as shown in the formula as follows:

(1) ASR=∑iri×wi∑iwi,

*i* is the age group; *r*_*i*_ is the age‐specific rate in the age group *i* (calculated as the number of events divided by the population size of that age group); *w*_*i*_ is the corresponding age‐specific weight in the GBD 2023 world standard population.

This study covered 31 countries and territories in the WPR, including China, Japan, Australia, New Zealand, the Republic of Korea, Singapore, the Philippines, Viet Nam, Cambodia, Malaysia, Mongolia, Papua New Guinea and several Pacific Island countries (the full list is provided in the Appendix Tables 2 and 3) [16].

This study was non‐clinical in nature. As the GBD database consists of publicly available, secondary, de‐identified data and does not involve individual‐level identifiable information, ethical approval and informed consent were not required. The study was conducted in accordance with the research guidelines of the GBD 2023 collaboration and represents findings produced by members of the collaboration [17].

### 2.3. Data Analysis

#### 2.3.1. Spatial Mapping and Temporal Trend Analysis

To describe the spatial distribution of RDs across countries and territories, maps of ASPR and ASDR per 100 000 population were generated [18]. Temporal trends in the burden of RDs from 1990 to 2023 were assessed using the estimated annual percentage change (EAPC). A log‐linear regression model was fitted as

(2) lnASR_t=α+βt+ε_t,

where *t* represents the calendar year and *β* represents the regression coefficient for year. The EAPC was calculated as

(3) EAPC=100×expβ−1.

The corresponding 95% confidence interval (CI) was derived from the 95% CI of *β* using the same transformation. An increasing trend was defined when both the EAPC estimate and the lower bound of its 95% CI were greater than 0, whereas a decreasing trend was defined when both the EAPC estimate and the upper bound of its 95% CI were below 0; otherwise, the trend was considered stable [19].

In addition, joinpoint regression models (Joinpoint Regression Program, version 4.9.0.0) were applied to identify significant inflection points in ASR trends and to estimate the annual percent change (APC) for each segment and the average annual percent change (AAPC) over the entire study period. Sex‐stratified analyses were also conducted to assess differences in temporal trends between males and females [20]. Given the descriptive and exploratory nature of the cross‐country, age‐, and sex‐specific comparisons, no additional correction for multiple comparisons was applied.

#### 2.3.2. Age–Sex Epidemiological Characteristics and Decomposition Analysis

Based on the GBD age‐group classifications, age‐specific prevalence rates and DALY rates were used to characterise the age‐specific distribution of RD burden, and differences between males and females across age groups were examined. In addition, a three‐factor decomposition analysis based on the Shapley decomposition framework was performed to partition changes in prevalent cases and DALYs from 1990 to 2023 into contributions from population growth, population ageing and epidemiological change [21]. The decomposition analysis was restricted to individuals aged 15 years and older, encompassing the 15–19‐year age group through the ≥ 95‐year age group. Accordingly, the overall differences reported in the decomposition analysis represent changes within this age range and are not directly comparable with the all‐age absolute differences reported elsewhere in the study. For each component, its contribution was estimated by averaging the marginal effect of changing that component across alternative sequences of changes in population size, population age structure and age‐specific rates. The resulting contributions from population growth, population ageing and epidemiological change sum algebraically to the observed net change within the analysed age range. For presentation of the decomposition results, the contribution of each component was additionally expressed relative to the corresponding burden in the baseline year (1990), calculated as the component contribution divided by the 1990 burden and multiplied by 100. The overall percentage change was calculated as the observed net change divided by the 1990 burden and multiplied by 100. Accordingly, the component percentages sum algebraically to the overall percentage change from baseline rather than to 100%. Sex‐stratified decomposition analyses were additionally conducted by applying the same framework separately to males and females.

#### 2.3.3. Forecasting of RD Burden to 2030

To estimate future trends in RD burden, annual ASPR and ASDR data from 1990 to 2023 were modelled using non‐seasonal autoregressive integrated moving average (ARIMA) models. Candidate ARIMA specifications were evaluated separately for each indicator and region, and the optimal model was selected based on the Akaike information criterion (AIC), corrected AIC (AICc), Bayesian information criterion (BIC) and residual diagnostics. Accordingly, model orders varied across regional time series; for the WPR, ARIMA (2,1,0) was selected for both ASPR and ASDR. The fitted models were used to project ASPR and ASDR through 2030, with point estimates and corresponding 95% prediction intervals (PIs) reported. Changes between the observed 2023 rates and projected 2030 rates were expressed as both absolute and percentage changes [22].

### 2.4. Statistical Software

All statistical analyses and graphical visualisations were conducted using R software. The main R packages used included ggplot2 (version 3.5.2), data.table (version 1.17.8) and dplyr (version 1.1.4). Joinpoint regression analyses were performed using the Joinpoint Regression Program (Version 4.9.0.0) provided by the US National Cancer Institute.

## 3. Results

### 3.1. Global and Regional Burden of RDs, 1990–2023

**FIGURE 1:**
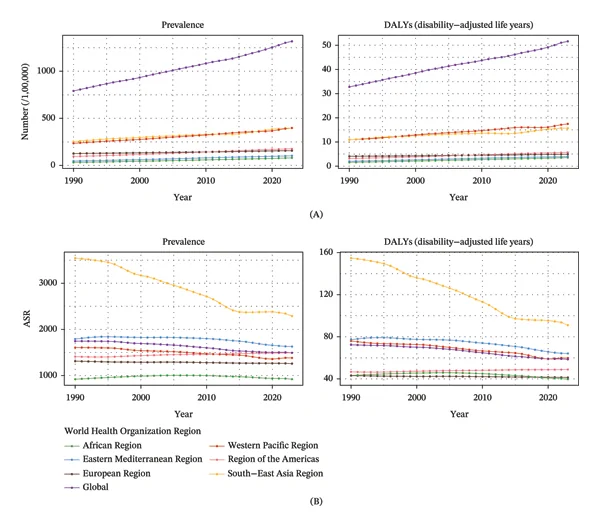
Global and regional burden of refractive disorders, 1990–2023. (A) Global and WHO regional RDs prevalence and DALY counts, 1990–2023. (B) Global and WHO regional RDs ASPR and ASDR, 1990–2023. Abbreviations: RDs, refractive disorders; DALYs, disability‐adjusted life years; ASPR, age‐standardised prevalence rate; ASDR, age‐standardised disability‐adjusted life years rate.

Between 1990 and 2023, the global number of prevalent cases of RDs increased steadily, whereas the ASPR declined overall (Figure 1A; Appendix Table 4). Globally, prevalent cases rose from 79.1 million (95% UI 71.6–86.9) in 1990 to 131.7 million (95% UI 118.9–145.6) in 2023. Over the same period, the global ASPR decreased from 1745.19 per 100,000 population to 1493.26 per 100,000 population, with EAPC of −0.56% (95% CI −0.59 to −0.53).

A similar pattern was observed for DALYs (Figure 1B; Appendix Table 5). The total number of DALYs increased from 3.28 million (95% UI 2.27–4.53) in 1990 to 5.16 million (95% UI 3.52–7.16) in 2023, while the ASDR declined from 72.36 per 100,000 population to 58.50 per 100,000 population (EAPC −0.73%, 95% CI −0.78 to −0.68).

Across WHO regions, the WPR and the South‐East Asia Region consistently bore the highest burden of RDs. In 2023, the South‐East Asia Region had the highest ASPR (2289.63 per 100,000 population; EAPC = −1.50%, 95% CI −1.58 to −1.42) and the highest ASDR (91.01 per 100,000 population; EAPC = −1.80%, 95% CI −1.88 to −1.71) and also experienced the fastest declines over time. In the WPR, the number of prevalent cases increased from 23.45 million in 1990 to 39.75 million in 2023, while the ASPR declined from 1606.27 per 100,000 population (95% UI 1454.97–1755.64) in 1990 to 1382.9 per 100,000 population (95% UI 1246.58–1519.73) in 2023 (EAPC = −0.53%, 95% CI −0.56 to −0.50). Similarly, DALYs increased from 1.09 million in 1990 to 1.75 million in 2023, accompanied by a significant decrease in the ASDR from 76 per 100,000 population (95% UI 53.63–101.94) in 1990 to 59.85 per 100,000 population (95% UI 41.91–81) in 2023 (EAPC = −0.78%, 95% CI −0.84 to −0.72).

In contrast, the Region of the Americas was the only WHO region in which both the ASPR (EAPC = 0.21%, 95% CI 0.19–0.22) and the ASDR (EAPC = 0.17%, 95% CI 0.16–0.19) showed increasing trends during the study period.

### 3.2. Regional Distribution and Temporal Trends of RDs in the WPR

**FIGURE 2:**
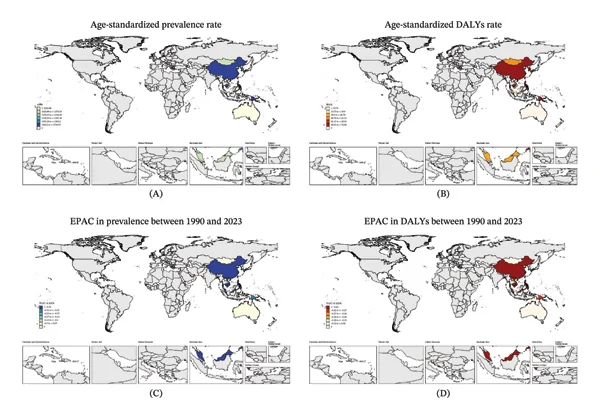
Global maps of refractive disorders ASPR, ASDR and EAPC across 31 Western Pacific countries. (A) World map of ASPR. (B) World map of ASDR. (C) EAPC in RDs prevalence, 1990–2023. (D) EAPC in RDs DALY rates, 1990–2023. Abbreviations: RDs, refractive disorders; EAPC, estimated annual percentage change; DALYs, disability‐adjusted life years; ASPR, age‐standardised prevalence rate; ASDR, age‐standardised disability‐adjusted life years rate.

Figure 2A,B shows marked spatial heterogeneity in ASPR and ASDR of RDs across countries in the WPR in 2023. Higher ASRs were mainly concentrated in East and South‐East Asia. China had relatively high ASPR and ASDR (1570.49 per 100,000 population [95% UI 1420.71–1723.89] and 70.83 per 100,000 population [95% UI 49.80–95.11]), as did the Republic of Korea (1365.20 per 100,000 population [95% UI 1211.82–1518.39] and 45.93 per 100,000 population [95% UI 30.82–68.88]). By contrast, Japan (828.06 per 100,000 population [95% UI 737.03–930.09] and 27.57 per 100,000 population [95% UI 18.39–41.33]) and Australia (888.53 per 100,000 population [95% UI 779.14–988.97] and 28.66 per 100,000 population [95% UI 19.25–43.19]) exhibited comparatively lower ASRs. Substantial heterogeneity was also observed among Pacific Island countries, with considerable variation in ASPR and ASDR across locations (Tables 1 and 2).

**TABLE 1:** Prevalence of refractive disorders in 31 Western Pacific countries from 1990 to 2023.

Location	1990 year		2023 year		EAPC (95 CI)
	Number (95% UI)	Rate (95% UI)	Number (95% UI)	Rate (95% UI)	
American Samoa	438.4 (388–493.03)	1409.2 (1236.1–1595.42)	656.62 (581.99–744.51)	1324.09 (1181.02–1493.59)	−0.18 (−0.19 to −0.17)
Australia	157056.19 (141682.37–176158.14)	884.64 (793.53–994.77)	294177.86 (260850.97–325788.41)	888.53 (795.14–988.97)	0.07 (0.02 to 0.12)
Brunei Darussalam	2465.72 (2128.16–2830.23)	1065.95 (950.47–1186.6)	4636.91 (4112.33–5248.79)	1020.88 (911.56–1149.1)	−0.13 (−0.14 to −0.12)
Cambodia	150613.08 (132223.24–171047.01)	2211.35 (1942.49–2502.74)	271549.19 (238531.87–305254.69)	1739.03 (1525.93–1955.39)	−1.32 (−1.55 to −1.1)
China	18285274.65 (16485407.1–20049842.72)	1904.76 (1720.74–2087.69)	30957203.17 (27422072.56–34337344.27)	1570.49 (1420.71–1723.89)	−0.7 (−0.74 to −0.66)
Cook Islands	203.29 (179.9–228.15)	1409.29 (1226.31–1579.73)	238.28 (207.71–271.75)	1309.05 (1148.84–1467.48)	−0.22 (−0.23 to −0.21)
Fiji	9211.23 (8217.77–10291.06)	1848.83 (1642.33–2066.73)	15079.83 (13441.82–16984.27)	1737.05 (1536.09–1958.14)	−0.11 (−0.29 to 0.08)
Guam	1378.44 (1229.41–1539.18)	1395.91 (1231.09–1567.45)	2592.74 (2258.18–2944.55)	1318.55 (1161.72–1481.43)	−0.17 (−0.18 to −0.16)
Japan	1127302.58 (1006649.63–1258650.89)	844.85 (756.54–948.37)	1382972.37 (1232574.7–1525423.22)	828.06 (737.03–930.09)	−0.05 (−0.06 to −0.04)
Kiribati	681.5 (593.61–764.74)	1431.23 (1253.17–1602.81)	1295.88 (1155.53–1456.67)	1364.96 (1197.33–1534.96)	−0.15 (−0.16 to −0.14)
Lao People’s Democratic Republic	26213.73 (23111.36–29594.05)	955.05 (841.85–1095.77)	55071.7 (48247.09–62935.78)	924.11 (802.36–1055.88)	−0.1 (−0.15 to −0.05)
Malaysia	200318.05 (184675.2–213449.95)	1365.25 (1268.28–1467.3)	355690.47 (309918.64–401189.14)	1033.89 (907.1–1164.89)	−1.04 (−1.21 to −0.87)
Marshall Islands	353.74 (313.98–393.66)	1442.58 (1279.19–1618.12)	425.09 (379.6–478.36)	1337.99 (1186.3–1501.89)	−0.22 (−0.23 to −0.21)
Micronesia (Federated States of)	897.64 (794.54–1002.58)	1441.64 (1274.37–1623.57)	1192.12 (1057.83–1349.87)	1355.35 (1196.41–1523.87)	−0.18 (−0.19 to −0.17)
Mongolia	18971.9 (16899.62–21310.46)	1329.03 (1176.65–1505.2)	38311.88 (33451.42–43295.53)	1274.19 (1124.19–1442.71)	−0.08 (−0.11 to −0.04)
Nauru	76.72 (67.87–86.27)	1412.84 (1249.96–1586.49)	105.8 (94.66–119.01)	1346.24 (1192.77–1508.87)	−0.14 (−0.16 to −0.12)
New Zealand	34079.69 (30217.96–37970.4)	942.62 (837.02–1054.05)	56302.59 (50272.08–62364.2)	921.15 (823.93–1027.4)	−0.07 (−0.07 to −0.07)
Niue	31.82 (28.01–35.57)	1410.63 (1249.08–1588.72)	28.69 (24.95–32.49)	1317.7 (1156.82–1480.88)	−0.2 (−0.21 to −0.19)
Northern Mariana Islands	388.05 (339.03–439.42)	1384.68 (1221.1–1557.27)	680.19 (598.41–778.41)	1319.95 (1172.04–1468.63)	−0.13 (−0.14 to −0.12)
Palau	161.7 (144.29–181.25)	1389.53 (1226.87–1554.65)	288.08 (249.72–331.51)	1310.96 (1148.62–1479.05)	−0.17 (−0.18 to −0.17)
Papua New Guinea	47145.52 (41399.04–53097.56)	1829.31 (1613.61–2057.83)	131673.08 (116533.34–149202.6)	1726.06 (1530.1–1948.84)	−0.22 (−0.27 to −0.18)
Philippines	603165.67 (535245.84–672103.12)	1481.01 (1321.1–1664.34)	1352303.12 (1202992.89–1523259.22)	1358.78 (1203.28–1527.23)	−0.32 (−0.42 to −0.22)
Republic of Korea	613943.86 (541020.88–687028.46)	1442.56 (1289.75–1590.24)	821803.77 (738415.64–908776.55)	1365.2 (1211.82–1518.39)	0.05 (−0.04 to 0.15)
Samoa	1639.09 (1453.44–1814.15)	1438.04 (1270.21–1615.42)	2496.42 (2218.18–2807.88)	1351.18 (1199.12–1522.53)	−0.18 (−0.2 to −0.17)
Singapore	32659.18 (29096.3–36861.85)	1111.95 (996.01–1235.47)	66830.44 (60020.23–74621.7)	1070.88 (958.77–1196.65)	−0.13 (−0.15 to −0.12)
Solomon Islands	2968.31 (2656.41–3313.85)	1472.42 (1311.36–1655.03)	8175.21 (7297.45–9176.91)	1390.17 (1228.83–1558)	−0.16 (−0.17 to −0.15)
Tokelau	21.31 (18.88–23.92)	1420.53 (1260.93–1595.4)	23.19 (20.38–26.25)	1315.69 (1155.83–1489.86)	−0.24 (−0.25 to −0.22)
Tonga	795.99 (707.08–891.39)	1190.35 (1049.65–1331.23)	972.96 (865.23–1092.94)	1076.67 (957.33–1215.56)	−0.31 (−0.34 to −0.27)
Tuvalu	108.83 (96.58–122.11)	1466.63 (1294.8–1649.9)	133.17 (116.9–151.02)	1360 (1195.31–1523.47)	−0.21 (−0.23 to −0.2)
Vanuatu	1018.32 (903.64–1125.65)	1094.24 (972.96–1230.95)	2549.02 (2272.14–2847.93)	1048.99 (926.64–1174.29)	−0.13 (−0.15 to −0.11)
Viet Nam	533776.81 (472775.92–590929.93)	1008.73 (898.19–1122.7)	1030375.85 (914396.21–1140527.72)	938.62 (833.59–1037.31)	−0.35 (−0.4 to −0.29)

*Note:* Prevalence of refractive disorders in 31 Western Pacific countries from 1990 to 2023.

Abbreviations: CI, confidence interval; EAPC, estimated annual percentage change; UI, uncertainty interval.

**TABLE 2:** DALYs of refractive disorders in 31 Western Pacific countries from 1990 to 2023.

Location	1990 year		2023 year		EAPC (95 CI)
	Number (95% UI)	Rate (95% UI)	Number (95% UI)	Rate (95% UI)	
American Samoa	14.53 (9.69–22.01)	44.39 (29.15–66.04)	20.24 (13.41–30.38)	40.66 (26.78–61.42)	−0.24 (−0.25 to −0.24)
Australia	5019.32 (3359.56–7612.94)	28.57 (19.17–43.01)	9090.02 (6035.35–13845.78)	28.66 (19.25–43.19)	0.06 (0.01 to 0.11)
Brunei Darussalam	86.69 (57.27–132.38)	36.05 (24.14–54.8)	154.26 (102.79–237.52)	34.11 (22.67–51.82)	−0.16 (−0.18 to −0.15)
Cambodia	6611.95 (4708.84–9222.72)	101.86 (73.07–138.21)	11279.2 (7887.21–15838.1)	72.68 (51.05–101.3)	−1.53 (−1.72 to −1.33)
China	894614.13 (639314.59–1215535.29)	94.65 (66.32–126.38)	1424688.19 (994146.98–1895696.28)	70.83 (49.8–95.11)	−0.97 (−1.04 to −0.9)
Cook Islands	6.47 (4.27–9.86)	43.82 (28.49–65.65)	7.15 (4.69–10.64)	39.71 (25.86–60)	−0.29 (−0.3 to −0.28)
Fiji	289.95 (193.39–443.27)	56.22 (37.02–84.94)	455.17 (301.48–690.12)	51.99 (34.12–79.95)	−0.15 (−0.33 to 0.03)
Guam	44.99 (29.29–66.88)	44.02 (28.27–66.42)	79.44 (50.5–119.59)	40.93 (26.56–61.69)	−0.22 (−0.22 to −0.21)
Japan	36783.13 (24508.59–55434.92)	28.19 (18.67–42.27)	42732.29 (27848.33–64317.08)	27.57 (18.39–41.33)	−0.06 (−0.07 to −0.05)
Kiribati	22.07 (14.59–33.22)	44.7 (29.15–68.03)	40.96 (26.97–62.28)	42.04 (27.38–63.9)	−0.18 (−0.19 to −0.17)
Lao People’s Democratic Republic	909.91 (607.62–1334.9)	32.96 (22.17–47.41)	1818.54 (1227.53–2711.86)	30.24 (20.38–45.07)	−0.25 (−0.3 to −0.2)
Malaysia	8478.07 (5801.39–12057.82)	58.24 (41.17–81.98)	13833.2 (9424.41–19500.37)	40.2 (27.44–56.27)	−1.29 (−1.46 to −1.12)
Marshall Islands	11.65 (7.54–17.51)	45.16 (29.5–67.46)	13.34 (8.9–20.06)	40.9 (26.86–60.41)	−0.29 (−0.3 to −0.27)
Micronesia (Federated States of)	29.29 (19.17–44.78)	45.09 (29.42–67.48)	37.01 (24.21–55.54)	41.31 (26.82–62.89)	−0.26 (−0.27 to −0.25)
Mongolia	635.94 (430.87–943.56)	42.87 (29.37–63.79)	1255.57 (869.33–1886.86)	41.09 (28.45–62.09)	−0.07 (−0.1 to −0.04)
Nauru	2.53 (1.66–3.85)	44.02 (28.77–64.99)	3.37 (2.19–5.13)	41.18 (26.81–61.22)	−0.19 (−0.21 to −0.17)
New Zealand	1093.47 (738.11–1653.32)	30.54 (20.64–46.15)	1755.51 (1171.05–2625.41)	29.87 (19.85–45.48)	−0.07 (−0.08 to −0.07)
Niue	0.99 (0.64–1.51)	44.05 (28.77–66.39)	0.87 (0.56–1.28)	40.32 (26.21–60.17)	−0.26 (−0.27 to −0.25)
Northern Mariana Islands	12.86 (8.22–19.38)	43.39 (27.91–64.77)	21.03 (13.65–31.85)	40.79 (26.31–60.87)	−0.17 (−0.17 to −0.16)
Palau	5.15 (3.46–7.85)	43.1 (28.52–64.69)	8.72 (5.69–13.16)	39.9 (25.92–60.47)	−0.23 (−0.24 to −0.23)
Papua New Guinea	1577 (1048.57–2346.43)	58.43 (39.66–89.25)	4259.78 (2821.47–6438.96)	54.11 (35.62–82.25)	−0.27 (−0.31 to −0.22)
Philippines	22243.42 (15286.79–32472.81)	54.03 (36.91–77.9)	47357.85 (32260.84–70363.57)	47.32 (32.21–69.31)	−0.43 (−0.54 to −0.33)
Republic of Korea	21633.12 (14277.45–32624.14)	49.48 (32.94–74.36)	26093.66 (17445.34–39482.34)	45.93 (30.82–68.88)	0.01 (−0.08 to 0.11)
Samoa	53.04 (35.12–81.51)	45.1 (29.79–68.72)	77.51 (51.72–116.96)	41.54 (27.45–62.37)	−0.24 (−0.25 to −0.23)
Singapore	1107.43 (727.91–1684.68)	37.36 (24.51–56.04)	2139.41 (1461.56–3231.32)	35.72 (24.35–52.33)	−0.16 (−0.17 to −0.14)
Solomon Islands	98.22 (65.21–148.1)	46.66 (31.23–70.51)	262.79 (176.91–403.79)	43.21 (28.42–65.37)	−0.21 (−0.22 to −0.21)
Tokelau	0.67 (0.43–0.99)	44.48 (28.99–65.44)	0.71 (0.45–1.06)	40.19 (25.81–60.4)	−0.31 (−0.32 to −0.3)
Tonga	26.83 (17.81–40.3)	38.8 (25.72–57.24)	30.89 (20.47–45.53)	33.75 (22.38–49.42)	−0.42 (−0.46 to −0.39)
Tuvalu	3.5 (2.34–5.27)	46.19 (30.51–70.09)	4.15 (2.75–6.29)	41.86 (27.37–63.79)	−0.28 (−0.29 to −0.27)
Vanuatu	33.17 (21.85–50.11)	34.23 (22.62–51.39)	80.77 (52.25–120.09)	32.44 (20.78–49.02)	−0.16 (−0.18 to −0.14)
Viet Nam	25063.97 (17651–34607.65)	48.71 (34.78–65.96)	43232.62 (30356.32–60347.42)	39.51 (27.64–55.36)	−0.74 (−0.83 to −0.65)

*Note:* DALYs of refractive disorders in 31 Western Pacific countries from 1990 to 2023.

Abbreviations: CI, confidence interval; EAPC, estimated annual percentage change; UI, uncertainty interval.

Between 1990 and 2023, the ASPR and ASDR declined in most countries in the WPR (Figure 2C,D). EAPC was negative in the majority of countries, indicating overall declines in ASRs. Only a small number of countries, including Australia (EAPC 0.06%, 95% CI 0.01–0.11) and the Republic of Korea (EAPC 0.01%, 95% CI −0.08–0.11), showed largely stable or slightly increasing ASRs.

Despite declining ASRs, the absolute numbers of prevalent cases and DALYs due to RDs increased in almost all countries in the WPR (Tables 1 and 2). China consistently accounted for the largest absolute burden in the region, with the number of prevalent cases increasing from approximately 18.29 million in 1990 to 30.96 million in 2023. DALYs increased from about 0.895 million in 1990 to 1.425 million in 2023; however, the ASPR declined over the study period (EAPC = −0.70%, 95% CI −0.74 to −0.66), and the ASDR also decreased substantially (EAPC = −0.97%, 95% CI −1.04 to −0.90). Countries such as Japan (27.57 per 100,000 population; EAPC = −0.06%, 95% CI −0.07 to −0.05), Singapore (35.72 per 100,000 population; EAPC = −0.24%, 95% CI −0.25 to −0.23) and Malaysia (40.20 per 100,000 population; EAPC = −1.29%, 95% CI −1.46 to −1.12) exhibited relatively low ASDRs overall, with comparatively modest changes over time.

### 3.3. Age–Sex Patterns of RDs in the WPR

**FIGURE 3:**
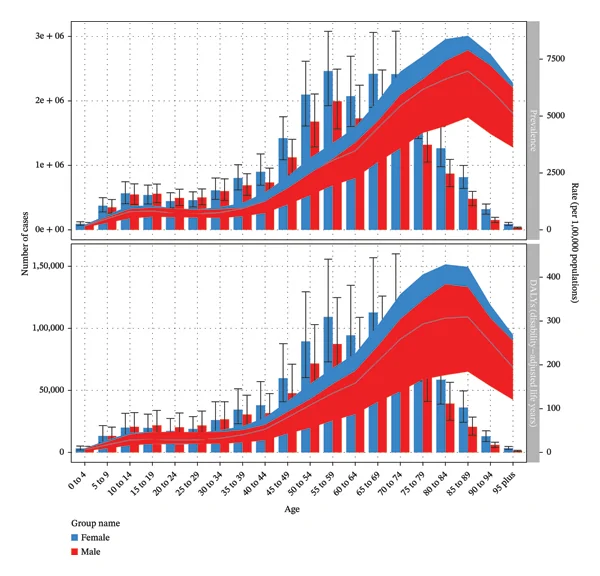
Age–sex epidemiological patterns of refractive disorders in the Western Pacific regions. Abbreviations: ASPR, age‐standardised prevalence rate; ASDR, age‐standardised disability‐adjusted life years rate.

As shown in Figure 3, prevalent cases and DALYs in the WPR in 2023 increased markedly with age, peaking in older adulthood before declining in the oldest age groups. Age‐specific prevalence and DALY rates also increased substantially after age 40 and were highest among individuals aged approximately 70–84 years. Females generally had higher numbers and age‐specific rates than males, with sex differences becoming more pronounced in middle and older ages. Between 1990 and 2023, age‐specific prevalence and DALY rates declined across most age–sex strata, with more pronounced declines in older age groups and relatively modest changes among children and adolescents. Despite these rate reductions, absolute prevalent cases and DALYs increased in most adult age groups (Appendix Tables 6 and 7).

### 3.4. Temporal Trends in RD Burden in the WPR

Figure 4 presents the joinpoint regression analyses of ASPR and ASDR for RDs in the WPR from 1990 to 2023. Overall, both indicators showed long‐term declining trends over the study period, with multiple significant inflection points. The AAPC in ASPR from 1990 to 2023 was −0.45% (95% CI −0.47 to −0.44). The ASPR declined slowly during 1990–1996 (APC = −0.13% [95% CI −0.2 to −0.03]), followed by more rapid decreases in 1996–1999 (APC = −1.06% [95% CI −1.22 to −0.8]), 1999–2004 (APC = −0.29% [95% CI −0.39 to −0.04]) and 2004–2016 (APC = −0.52% [95% CI −0.56 to −0.49]). The steepest decline occurred during 2016–2019 (APC = −1.65% [95% CI −1.79 to −1.37]), after which a slight increase was observed in 2019–2023 (APC = 0.42% [95% CI 0.27–0.56]) (Figure 4A). Similar segmented trends were observed in both sexes, with a more pronounced decline among males during 2016–2019 (APC = −1.75% [95% CI −1.93 to −1.38]) (Figure 4B,C).

FIGURE 4Joinpoint analysis and trend patterns of refractive disorders by sex, 1990–2023. (A) Prevalence for both with RDs in WPR. (B) Prevalence for male with RDs in WPR. (C) Prevalence for female with RDs in WPR. (D) DALYs for both with RDs in WPR. (E) DALYs for male with RDs in WPR. (F) DALYs for female with RDs in WPR. (G) Trends in sex‐specific RDs prevalence counts. (H) Trends in sex‐specific RDs DALYs counts. Abbreviations: RDs, refractive disorders; DALYs, disability‐adjusted life years; ASPR, age‐standardised prevalence rate; ASDR, age‐standardised disability‐adjusted life years rate; WPR, Western Pacific region; APC, annual percent change; AAPC, average annual percent change.

**Figure:**
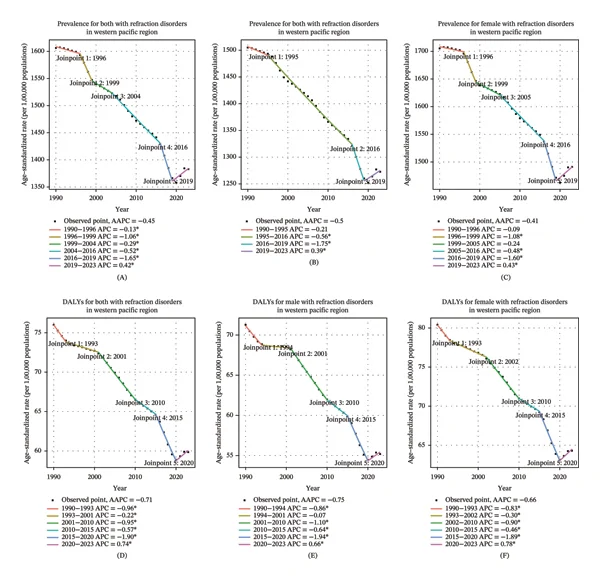

**Figure:**
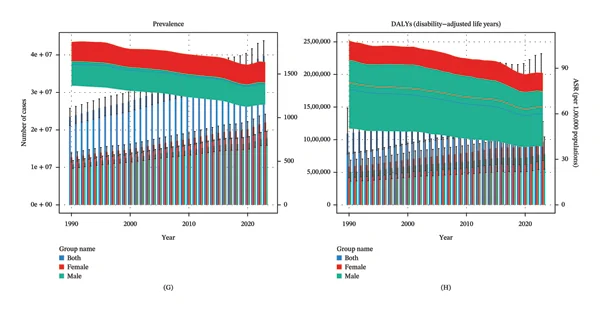

From 1990 to 2023, AAPC in ASDR was −0.71% (95% CI −0.73 to −0.69). Overall, ASDR showed a declining trend over the study period, with particularly pronounced reductions during 1990–1993 (APC = −0.96% [95% CI −1.34 to −0.72]) and 2015–2020 (APC = −1.90% [95% CI −2 to −1.8]), followed by a modest increase after 2020 (APC = 0.74% [95% CI 0.56 to 0.92]) (Figure 4D). Despite the overall declines in ASRs, the absolute numbers of prevalent cases and DALYs for RDs increased continuously in both sexes throughout the study period (Figure 4G,H). Detailed results are provided in Appendix Tables 8 and 9.

### 3.5. Decomposition of Changes in RD Burden in the WPR

**FIGURE 5:**
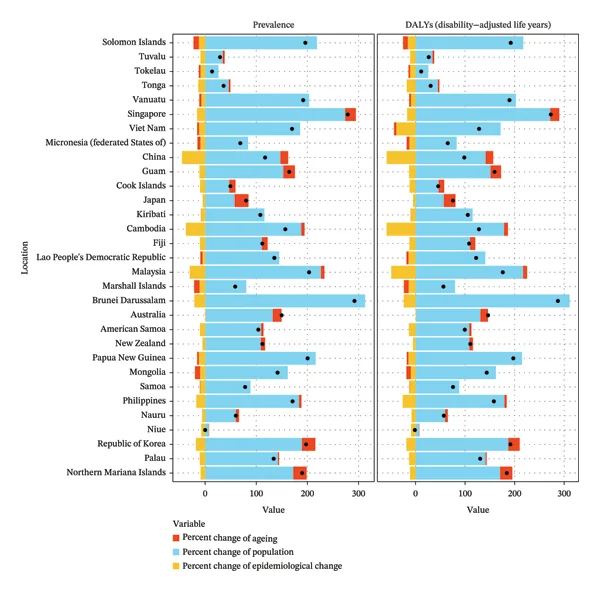
Decomposition of changes in refractive disorder burden in 31 Western Pacific countries.

Figure 5 illustrates the decomposition of changes in prevalent cases and DALYs due to RDs among individuals aged ≥ 15 years from 1990 to 2023 across 31 countries and territories in the WPR into contributions from population growth, population ageing and epidemiological change. For prevalent cases, population growth was the dominant positive contributor in most countries, with particularly large absolute contributions in China (16.83 million cases), the Philippines (0.60 million) and Viet Nam (0.46 million). Population ageing also contributed substantially in several countries, particularly in East Asia, whereas epidemiological change was negative in most settings and partly offset demographic increases.

A similar pattern was observed for DALYs. Population growth remained the major positive contributor, including approximately 0.83 million DALYs in China, while population ageing also contributed substantially in several East Asian countries. Negative epidemiological changes partly offset these demographic effects in most countries. Detailed absolute Shapley contributions and their percentage changes relative to the 1990 baseline burden are presented in Appendix Table 10.

Sex‐stratified decomposition showed broadly similar patterns between males and females. However, population ageing generally made a larger absolute contribution to female than male burden, particularly in East Asia. Increases in prevalent cases and DALYs were also generally greater among females, while epidemiological change remained negative in nearly all settings and partly offset demographic increases (Appendix Table 11).

### 3.6. Projected Burden of RDs to 2030

**FIGURE 6:**
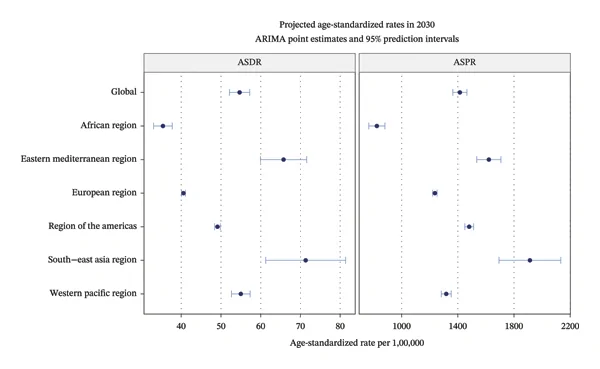
Projected age‐standardised prevalence and DALY rates of refractive disorders in 2030 globally and across WHO regions. ARIMA models were used to project age‐standardised rates to 2030. Points represent projected estimates, and horizontal bars represent 95% prediction intervals. Rates are expressed per 1,00,000 population. Abbreviations: ASPR, age‐standardised prevalence rate; ASDR, age‐standardised disability‐adjusted life years rate.

ARIMA projections suggested that age‐standardised RD rates would continue to decline in the WPR through 2030 (Figure 6; Appendix Table 12). The ASPR was projected to decrease from 1382.90 per 100,000 population in 2023 to 1317.70 per 100,000 (95% PI 1282.64–1352.76) in 2030, corresponding to a 4.71% reduction. The ASDR was projected to decline from 59.85 to 55.00 per 100,000 (95% PI 52.66–57.34), representing an 8.10% reduction. Globally, ASPR and ASDR were similarly projected to decline by 5.26% and 6.51%, respectively, although substantial regional heterogeneity remained, with South‐East Asia projected to retain the highest age‐standardised burden in 2030.

## 4. Discussion

### 4.1. Declining ASRs and Persistent Public Health Pressure

The sustained decline in ASRs occurred alongside improvements in eye health systems in parts of the WPR, including the expanded availability of refractive examinations and correction technologies, strengthened primary eye‐care capacity and the implementation of routine vision screening programmes for children and adolescents in some countries [23]. These advances are consistent with the WHO target of increasing effective coverage for RDs [24]. However, our findings indicate that declining ASRs have not been accompanied by a proportional reduction in absolute burden, while decomposition analysis showed substantial contributions from population growth and ageing. The WPR is undergoing one of the world’s most rapid population‐ageing processes, especially in populous countries including China [25]. In this setting, demographic changes stemming from a large population base have coincided with sustained increases in RDs. Given that RD burden increases markedly with age, population‐level prevalent cases and DALYs keep increasing even as ASRs decline [26]. This pattern highlights the limitations of relying solely on age‐standardised indicators and underscores the need, particularly in settings experiencing rapid demographic transition, to incorporate absolute burden metrics into eye health planning and resource allocation to better address growing service demands [27].

### 4.2. Age‐ and Sex‐Specific Patterns of RD Burden

Our findings showed that declines in age‐specific rates were more pronounced among older adults, whereas changes among children and adolescents were relatively modest. Older adults had a substantially higher baseline burden, consistent with the accumulation of RDs across the life course [28, 29], while improvements in access to refractive correction and eye‐care services may have contributed to the observed declines [30, 31]. In younger populations, persistent educational intensity, near‐work and lifestyle‐related exposures may partly explain the more modest changes, although these factors were not directly assessed and should not be interpreted causally [16, 26].

Females consistently had higher numbers of prevalent cases and DALYs across most age groups. Sex‐stratified decomposition further showed that population ageing contributed more strongly to increases in female burden in most countries, particularly in East Asia. Longer female life expectancy and the greater representation of women in older age groups may partly explain this disparity [32, 33]. Biological and gender‐related factors, including hormonal influences, near‐work exposure, health‐care utilisation and access to refractive correction, may also be relevant [33, 34], but their relative contributions could not be determined in this study. These findings highlight the importance of considering older women in refractive service planning [34].

### 4.3. Non‐Linear Temporal Trends and Cross‐National Heterogeneity

The segmented trends occurred within a changing policy and health‐system context in the WPR. Fluctuations during 1990–2004 were accompanied by heterogeneous socioeconomic and health‐system changes, whereas the decline during 2004–2016 overlapped with increasing regional emphasis on universal eye health, including the WHO Western Pacific Regional Action Plan for Universal Eye Health (2014–2019). The steeper decline during 2016–2019 also coincided with increasing policy attention to refractive health, including China’s national childhood myopia prevention programme introduced in 2018 [35]. These temporal correspondences, however, do not establish causal effects of specific policies.

The slight rebound after 2019 coincided with the COVID‐19 period, during which increased near‐work and screen exposure, reduced outdoor activity and accelerated myopia progression were widely reported [36]. Changes in health‐care utilisation, case ascertainment and other behavioural or health‐system factors may also have contributed [36]. Distinguishing among these explanations would require additional data on service utilisation, behavioural exposures, detection practices and country‐specific policy changes; therefore, the rebound should be interpreted as a temporal association rather than evidence of a specific causal mechanism.

Under the Shapley decomposition framework, the effects of population growth, population ageing and epidemiological change are jointly estimated, allowing positive and negative components to offset one another. Accordingly, an absolute component contribution may exceed the observed net change when offset by an opposing component, while its baseline‐relative percentage may exceed 100%. Negative epidemiological contributions should be interpreted as indicating that changes in age‐specific RD rates partly counterbalanced demographic pressures, rather than as evidence of a negative disease burden or the effect of any specific intervention. Cross‐country differences should also be interpreted in the context of variation in eye‐care capacity and access to refractive services [9], as well as educational, near‐work and other environmental exposures relevant to refractive error [2, 7]. In particular, relatively low modelled ASRs in some Pacific Island countries should not be equated with low unmet need because sparse primary data, limited surveillance and under‐detection may influence GBD estimates.

These findings support context‐specific strategies for RD control. Although our analysis addresses RDs as an aggregate category, intervention studies from the WPR support school‐based vision screening linked to timely and affordable spectacle provision, together with increased outdoor time for childhood myopia prevention [37–39]. Although ARIMA projections suggest further declines in ASPR and ASDR in the WPR by 2030, this does not indicate that the SPECS 2030 target will necessarily be achieved because the target concerns a 40‐percentage‐point increase in the effective refractive error coverage rather than reductions in disease rates alone [40]. Continued demographic pressure therefore underscores the need to expand affordable refractive correction, strengthen primary eye‐care capacity and improve monitoring of effective coverage.

### 4.4. Strengths and Limitations

A key strength of this study is the integration of long‐term temporal trends, age‐ and sex‐specific analyses, demographic decomposition and forecasting, enabling a comprehensive assessment of RD burden in the WPR. Several limitations should be acknowledged. First, GBD estimates depend on the availability and quality of underlying data, and limited surveillance, refractive equipment and trained personnel may contribute to under‐detection in resource‐constrained settings [14]. Second, GBD 2023 reports RDs as an aggregate category; therefore, subtype‐specific analyses were not feasible, and aggregation may mask differences among myopia, hyperopia, astigmatism, presbyopia and other refractive or accommodation disorders [41]. Third, harmonised country‐level data on health‐system characteristics, educational environments, urbanisation and near‐work or screen‐time exposure were unavailable, limiting interpretation of cross‐country heterogeneity. Finally, ARIMA projections extrapolate historical trends without incorporating future changes in demography, policy, health‐service coverage or behavioural exposures and should therefore be interpreted as trend‐based rather than deterministic forecasts. The absence of individual‐level risk‐factor data further limits causal inference [29].

## 5. Conclusion

Despite long‐term declines in ASPR and ASDR, the absolute burden of RDs increased substantially in the WPR from 1990 to 2023. Population growth and ageing were important demographic contributors to this increase, while the greater ageing‐related contribution among females further highlights the need to consider age and sex differences in eye‐health planning. Although ASPR and ASDR are projected to decline further by 2030, these trends do not necessarily imply a reduction in future service demand or achievement of the SPECS 2030 target. Monitoring ASRs together with absolute burden and effective refractive error coverage, while strengthening accessible and equitable refractive services, will be essential for addressing the evolving RD burden in the WPR.

## Author Contributions

Yuqi Jiang and Yalin Wu conceived the research idea. Yuqi Jiang, Shinan Wu, Yuling Chen and Jinzhi Peng conducted data cleaning and literature review. Yuqi Jiang and Yalin Wu contributed to drafting and critically revising the work for intellectual content. Yuqi Jiang and Shinan Wu conducted the analysis and created the figures and tables. Zuguo Liu and Yalin Wu provided a critical review of the manuscript.

## Funding

This work was supported by the National Key R&D Program of China (grant no. 2024YFA1108700), the National Natural Science Foundation of China (grant no. 82471093) and the Shenzhen Science and Technology Program (grant no. JCYJ20230807091503007).

## Disclosure

The funders had no role in study design. All authors have read and approved the manuscript.

## Ethics Statement

The data for this study were obtained from the publicly available dataset, the Global Burden of Disease (GBD) Database. The GBD data are thoroughly de‐identified and contain no personally identifiable information. Therefore, ethical approval and consent were not required.

## Consent

The authors have nothing to report.

## Conflicts of Interest

The authors declare no conflicts of interest.

## Supporting Information

Additional supporting information can be found online in the Supporting Information section.

## Supporting information


**Supporting Information** The Supporting Appendix contains 12 tables detailing refractive disorder definitions, SDI classifications for the 31 Western Pacific Region countries and territories, global and WHO regional prevalence and DALY estimates, age‐ and sex‐specific burden, joinpoint regression results, Shapley decomposition analyses and ARIMA projections to 2030.

## Data Availability

The datasets presented in this study can be found in online repositories. The names of the repository/repositories and accession number(s) can be found in GBD Publish Dashboard (https://vizhub.healthdata.org/gbd-results/).
