# Supplementary material for: Changing Epidemiology of Refractive Disorders in the Western Pacific Region, 1990–2023: Evidence From the Global Burden of Disease Study 2023
Source: J Ophthalmol. 2026 Sep 24;2026:1397428. doi: 10.1155/joph/1397428 (PMC13602173; doi:10.1155/joph/1397428)
Supplement: Supplementary file 1 [file JOPH-2026-1397428-s001.docx]

**Changing Epidemiology of Refractive Disorders in the Western Pacific Region, 1990-2023: Evidence from the Global Burden of Disease Study 2023**

**Appendix Table 1. Distribution of Disease Definitions for Refractive Disorders**

| Cause ID | Cause Hierarchy Level | Cause Name | ICD10 | ICD10 Used in Hospital/Claims Analyses | ICD9 | ICD9 Used in Hospital/Claims Analyses |
| --- | --- | --- | --- | --- | --- | --- |
| 999 | 4 | Refraction disorders | H52-H52.7 |  | 367-367.9 |  |

**Appendix Table 2. SDI Category Definitions and Corresponding Index Ranges**

| **location_id** | **location_name** | **lower_bound** | **upper_bound** |
| --- | --- | --- | --- |
| 44637 | **Low SDI** | 0 | 0.5315638184893383 |
| 44636 | **Low-middle SDI** | 0.5315638184893383 | 0.6304845023962745 |
| 44639 | **Middle SDI** | 0.6304845023962745 | 0.6766624946759122 |
| 44634 | **High-middle SDI** | 0.6766624946759122 | 0.718826599477871 |
| 44635 | **High SDI** | 0.718826599477871 | 1 |

| **Appendix Table 3. The 31 Countries and Their SDI Levels in the Western Pacific Region** | | | | |
| --- | --- | --- | --- | --- |
| location_name | mean_value | lower_value | upper_value | SDI |
| Republic of Korea | 0.891662727 | 0.891662727 | 0.891662727 | High SDI |
| Japan | 0.875884075 | 0.875884075 | 0.875884075 | High SDI |
| Singapore | 0.869993868 | 0.869993868 | 0.869993868 | High SDI |
| New Zealand | 0.866430611 | 0.866430611 | 0.866430611 | High SDI |
| Australia | 0.855660217 | 0.855660217 | 0.855660217 | High SDI |
| Brunei Darussalam | 0.832363947 | 0.832363947 | 0.832363947 | High SDI |
| Guam | 0.811868521 | 0.811868521 | 0.811868521 | High SDI |
| Northern Mariana Islands | 0.791104245 | 0.791104245 | 0.791104245 | High SDI |
| Cook Islands | 0.777404869 | 0.777404869 | 0.777404869 | High SDI |
| Malaysia | 0.770371353 | 0.770371353 | 0.770371353 | High SDI |
| Palau | 0.75589719 | 0.75589719 | 0.75589719 | High SDI |
| Tokelau | 0.749948959 | 0.749948959 | 0.749948959 | High SDI |
| Niue | 0.748490237 | 0.748490237 | 0.748490237 | High SDI |
| American Samoa | 0.743030595 | 0.743030595 | 0.743030595 | High SDI |
| China | 0.727553274 | 0.727553274 | 0.727553274 | High SDI |
| Fiji | 0.686418864 | 0.686418864 | 0.686418864 | High-middle SDI |
| Philippines | 0.669416191 | 0.669416191 | 0.669416191 | Middle SDI |
| Mongolia | 0.659697833 | 0.659697833 | 0.659697833 | Middle SDI |
| Viet Nam | 0.64302812 | 0.64302812 | 0.64302812 | Middle SDI |
| Tonga | 0.63599479 | 0.63599479 | 0.63599479 | Middle SDI |
| Nauru | 0.626371926 | 0.626371926 | 0.626371926 | Low-middle SDI |
| Tuvalu | 0.611233842 | 0.611233842 | 0.611233842 | Low-middle SDI |
| Marshall Islands | 0.605458023 | 0.605458023 | 0.605458023 | Low-middle SDI |
| Samoa | 0.604489559 | 0.604489559 | 0.604489559 | Low-middle SDI |
| Micronesia (Federated States of) | 0.596840305 | 0.596840305 | 0.596840305 | Low-middle SDI |
| Kiribati | 0.542688753 | 0.542688753 | 0.542688753 | Low-middle SDI |
| Lao People's Democratic Republic | 0.515008269 | 0.515008269 | 0.515008269 | Low SDI |
| Cambodia | 0.499953382 | 0.499953382 | 0.499953382 | Low SDI |
| Vanuatu | 0.47654316 | 0.47654316 | 0.47654316 | Low SDI |
| Solomon Islands | 0.45802266 | 0.45802266 | 0.45802266 | Low SDI |
| Papua New Guinea | 0.43671418 | 0.43671418 | 0.43671418 | Low SDI |

| **Appendix Table 4. Age-standardised prevalence of refractive disorders in global and WHO regions from 1990 to 2023** | | | | | |
| --- | --- | --- | --- | --- | --- |
| Location | 1990 Year | | 2023 Year | | EAPC (95CI) |
|  | Number (95%UI; million) | ASPR per 100,000 (95% UI) | Number (95%UI; million) | ASPR per 100,000 (95% UI) |  |
| Global | 79.08 (71.59 to 86.86) | 1745.19 (1587.87 to 1916.09) | 131.73 (118.95 to 145.64) | 1493.26 (1353.95 to 1646.84) | -0.56(-0.59 to -0.53) |
| European Region | 12.61 (11.42 to 13.90) | 1312.37 (1188.56 to 1438.95) | 15.67 (14.11 to 17.36) | 1259.46 (1139.23 to 1385.81) | -0.11(-0.12 to -0.1) |
| Eastern Mediterranean Region | 4.59 (4.15 to 5.07) | 1791.85 (1624.71 to 1968.61) | 10.25 (9.28 to 11.30) | 1629.2 (1475.94 to 1802.55) | -0.33(-0.4 to -0.26) |
| Region of the Americas | 9.55 (8.57 to 10.59) | 1410.44 (1269.09 to 1560.74) | 17.67 (15.95 to 19.56) | 1493 (1343.36 to 1645.7) | 0.21(0.19 to 0.22) |
| African Region | 3.20 (2.87 to 3.56) | 921.11 (834.03 to 1014.42) | 8.02 (7.15 to 8.93) | 921.38 (827.59 to 1026.85) | -0.01(-0.11 to 0.09) |
| South-East Asia Region | 25.28 (22.65 to 28.13) | 3543.67 (3164.27 to 3957.49) | 39.63 (35.30 to 44.56) | 2289.63 (2037.46 to 2570.94) | -1.5(-1.58 to -1.42) |
| Western Pacific Region | 23.45 (21.16 to 25.75) | 1606.27 (1454.97 to 1755.64) | 39.75 (35.53 to 43.78) | 1382.9 (1246.58 to 1519.73) | -0.53(-0.56 to -0.5) |

**Abbreviations:** CI, Confidence interval; UI, Uncertainty Interval; EAPC, Estimated Annual Percentage Change; WHO, World Health Organization Region; ASPR, age-standardised prevalence rates

**Notes:**Rates presented in this table are ASPR calculated using the GBD world standard population.

| **Appendix Table 5. DALYs of refractive disorders in global and WHO regions from 1990 to 2023** | | | | | |
| --- | --- | --- | --- | --- | --- |
| Location | 1990 Year | | 2023 Year | | EAPC (95CI) |
|  | Number (95%UI; million) | Rate (95%UI) | Number (95%UI; million) | Rate (95%UI) |  |
| Global | 3.28 (2.27 to 4.53) | 72.36 (50.32 to 98.98) | 5.16 (3.52 to 7.16) | 58.5 (39.85 to 81.42) | -0.73(-0.78 to -0.68) |
| African Region | 0.16 (0.11 to 0.21) | 43.2 (30.19 to 58.13) | 0.35 (0.25 to 0.49) | 39.67 (27.47 to 54.59) | -0.27(-0.39 to -0.16) |
| Region of the Americas | 0.32 (0.21 to 0.48) | 46.64 (31.26 to 69.81) | 0.57 (0.38 to 0.85) | 48.89 (32.71 to 73.22) | 0.17(0.16 to 0.19) |
| European Region | 0.41 (0.28 to 0.61) | 43.13 (29.51 to 64.39) | 0.49 (0.33 to 0.74) | 41.28 (28.16 to 62.09) | -0.11(-0.12 to -0.1) |
| Western Pacific Region | 1.09 (0.78 to 1.48) | 76 (53.63 to 101.94) | 1.75 (1.23 to 2.32) | 59.85 (41.91 to 81) | -0.78(-0.84 to -0.72) |
| South-East Asia Region | 1.09 (0.76 to 1.49) | 154.69 (108.93 to 207.25) | 1.57 (1.07 to 2.15) | 91.01 (62.22 to 124.82) | -1.8(-1.88 to -1.71) |
| Eastern Mediterranean Region | 0.20 (0.14 to 0.28) | 77.26 (52.88 to 106.46) | 0.40 (0.27 to 0.57) | 64.11 (43.44 to 90.52) | -0.63(-0.71 to -0.54) |

**Abbreviations:** CI, Confidence interval; UI, Uncertainty Interval; EAPC, Estimated Annual Percentage Change; WHO, World Health Organization Region

**Notes:**DALYs of Refractive disorders in global and WHO regions from 1990 to 2023

| **Appendix Table 6. Age- and sex-specific prevalence of refractive disorders in the Western Pacific Region from 1990 to 2023** | | | | | | |
| --- | --- | --- | --- | --- | --- | --- |
| Sex | Age | 1990 Year | | 2023 Year | | EAPC (95%CI) |
|  |  | Number (95%UI) | Prevalence rate per 100,000 (95% UI) | Number (95%UI) | Prevalence rate per 100,000 (95% UI) |  |
| Both | 0 to 4 | 259606.73 (193039.01 ~ 347144.48) | 152.71 (113.55 ~ 204.21) | 178641.95 (133017.95 ~ 239915.52) | 153.14 (114.03 ~ 205.67) | 0.01(-0.03 to 0.06) |
|  | 10 to 14 | 1231560.59 (892403.9 ~ 1598408.34) | 770.85 (558.57 ~ 1000.46) | 1114322.03 (796883.04 ~ 1457376.94) | 749.76 (536.17 ~ 980.58) | 0(-0.18 to 0.18) |
|  | 15 to 19 | 1517109.91 (1146806.43 ~ 1918960.37) | 835.66 (631.69 ~ 1057.01) | 1099485.82 (820313.05 ~ 1403432.96) | 794.81 (593 ~ 1014.53) | -0.09(-0.26 to 0.08) |
|  | 20 to 24 | 1416219.41 (1091178.6 ~ 1807797.69) | 781.15 (601.86 ~ 997.13) | 940271.03 (718135.61 ~ 1211034.29) | 729.69 (557.3 ~ 939.81) | -0.22(-0.31 to -0.12) |
|  | 25 to 29 | 1176852.28 (912991.29 ~ 1474300.44) | 758.93 (588.77 ~ 950.75) | 963948.87 (744665.95 ~ 1230911.84) | 699.15 (540.11 ~ 892.78) | -0.25(-0.33 to -0.18) |
|  | 30 to 34 | 1010574.97 (778133.3 ~ 1327998.3) | 784.97 (604.42 ~ 1031.53) | 1212397.08 (931704.2 ~ 1597269.23) | 722.26 (555.04 ~ 951.54) | -0.23(-0.31 to -0.15) |
|  | 35 to 39 | 1166214.26 (906588.07 ~ 1467709.33) | 919.77 (715.01 ~ 1157.55) | 1496133.24 (1155980.41 ~ 1902424.89) | 850.14 (656.86 ~ 1081.01) | -0.2(-0.27 to -0.14) |
|  | 40 to 44 | 1165299.66 (888852.09 ~ 1512819.23) | 1185.68 (904.4 ~ 1539.28) | 1635381.82 (1246916.32 ~ 2141107.67) | 1114.07 (849.43 ~ 1458.58) | -0.16(-0.23 to -0.1) |
|  | 45 to 49 | 1344929.25 (1027300.39 ~ 1676994.19) | 1730.99 (1322.19 ~ 2158.38) | 2547521.92 (1947127.52 ~ 3159685.22) | 1680.4 (1284.37 ~ 2084.2) | -0.08(-0.16 to 0) |
|  | 5 to 9 | 763079.7 (564555.39 ~ 1012978.72) | 465.08 (344.09 ~ 617.39) | 724519.64 (538472.22 ~ 970359.68) | 452.75 (336.49 ~ 606.37) | 0.01(-0.08 to 0.09) |
|  | 50 to 54 | 1694872.11 (1300981.38 ~ 2096824.46) | 2392.97 (1836.84 ~ 2960.49) | 3776319.65 (2883242.32 ~ 4715236.52) | 2255.93 (1722.41 ~ 2816.83) | -0.17(-0.23 to -0.11) |
|  | 55 to 59 | 2057046.39 (1624644.05 ~ 2594120.66) | 3235.81 (2555.62 ~ 4080.65) | 4457498.08 (3493456.1 ~ 5588699.87) | 2774.04 (2174.09 ~ 3478.03) | -0.51(-0.55 to -0.46) |
|  | 60 to 64 | 2173344.15 (1705984.54 ~ 2763771.75) | 4170.76 (3273.87 ~ 5303.82) | 3802560.24 (2930690.13 ~ 4955126.99) | 3216.95 (2479.35 ~ 4192.01) | -0.85(-0.9 to -0.8) |
|  | 65 to 69 | 2247560.14 (1796077.2 ~ 2781465.08) | 5630.68 (4499.6 ~ 6968.24) | 4389684.15 (3449609.43 ~ 5515092.97) | 4140.83 (3254.05 ~ 5202.44) | -1.04(-1.11 to -0.98) |
|  | 70 to 74 | 1908165.1 (1500819.73 ~ 2392398.93) | 6912.87 (5437.15 ~ 8667.15) | 4282111.82 (3333318.54 ~ 5454128.7) | 5051.59 (3932.3 ~ 6434.22) | -1.18(-1.26 to -1.1) |
|  | 75 to 79 | 1297741.66 (1053654.36 ~ 1585726.97) | 7395.65 (6004.63 ~ 9036.84) | 3096217.86 (2469533.83 ~ 3831893.64) | 5789.38 (4617.59 ~ 7164.97) | -1.08(-1.19 to -0.97) |
|  | 80 to 84 | 684499.4 (535812.45 ~ 850216.79) | 7474.79 (5851.11 ~ 9284.44) | 2136811.06 (1642699.01 ~ 2679614.69) | 6318.19 (4857.18 ~ 7923.17) | -0.87(-0.97 to -0.76) |
|  | 85 to 89 | 269267.65 (211467.74 ~ 328973.26) | 7385.13 (5799.87 ~ 9022.66) | 1296884.05 (1015802.35 ~ 1590569.67) | 6732.96 (5273.69 ~ 8257.68) | -0.67(-0.8 to -0.55) |
|  | 90 to 94 | 57046.3 (43481.69 ~ 70927.9) | 6069.17 (4626.03 ~ 7546.04) | 471769.11 (356018.23 ~ 595496.69) | 5985.36 (4516.82 ~ 7555.1) | -0.39(-0.52 to -0.27) |
|  | 95 plus | 9936.4 (7461.61 ~ 12600.41) | 5247.51 (3940.55 ~ 6654.4) | 125158.39 (93542.92 ~ 158407.83) | 5045.68 (3771.12 ~ 6386.11) | -0.3(-0.4 to -0.21) |
|  | All ages | 23450926.08 (21163860.78 ~ 25750221.28) | 1356.51 (1224.22 ~ 1489.51) | 39747637.83 (35531149.82 ~ 43776930.22) | 1785.13 (1595.76 ~ 1966.09) | 0.76(0.73 to 0.79) |
| Female | 0 to 4 | 137401.93 (101480.64 ~ 183217.04) | 169.24 (124.99 ~ 225.67) | 93252.76 (69249.46 ~ 124520.76) | 168.47 (125.11 ~ 224.96) | -0.01(-0.05 to 0.03) |
|  | 10 to 14 | 640651.83 (468496.06 ~ 831140.22) | 825.86 (603.94 ~ 1071.42) | 565565.04 (405105.79 ~ 746428.33) | 801.57 (574.15 ~ 1057.9) | -0.03(-0.21 to 0.14) |
|  | 15 to 19 | 774076.01 (580917.53 ~ 977725.3) | 870.52 (653.29 ~ 1099.54) | 540206.99 (401019.12 ~ 693811.37) | 825 (612.44 ~ 1059.59) | -0.14(-0.32 to 0.04) |
|  | 20 to 24 | 709964.14 (542613.24 ~ 903003) | 793.61 (606.54 ~ 1009.39) | 446081.84 (340757.91 ~ 575533.93) | 728.82 (556.74 ~ 940.32) | -0.28(-0.4 to -0.15) |
|  | 25 to 29 | 593834.16 (463200.25 ~ 741724.24) | 776.37 (605.58 ~ 969.72) | 460725 (356210.71 ~ 589743.52) | 702.71 (543.3 ~ 899.5) | -0.3(-0.41 to -0.2) |
|  | 30 to 34 | 519044.86 (401551.36 ~ 684547.29) | 829.72 (641.9 ~ 1094.28) | 611504.74 (472951.31 ~ 803551.99) | 760.65 (588.3 ~ 999.53) | -0.24(-0.36 to -0.12) |
|  | 35 to 39 | 628692.81 (488741.01 ~ 791910.52) | 1017.56 (791.04 ~ 1281.73) | 804047.87 (619772.56 ~ 1011308.89) | 938.88 (723.7 ~ 1180.89) | -0.22(-0.32 to -0.11) |
|  | 40 to 44 | 635393.78 (485381.25 ~ 825297.73) | 1338.41 (1022.42 ~ 1738.43) | 900653.07 (691874.21 ~ 1177113.06) | 1256.82 (965.48 ~ 1642.6) | -0.16(-0.25 to -0.07) |
|  | 45 to 49 | 730244.3 (559533.79 ~ 904966.53) | 1943.44 (1489.12 ~ 2408.44) | 1422678.87 (1086906.83 ~ 1754227.12) | 1903.19 (1454.01 ~ 2346.72) | -0.05(-0.13 to 0.04) |
|  | 5 to 9 | 403426.83 (300396.66 ~ 534134.71) | 509.59 (379.45 ~ 674.69) | 374352.73 (278673.43 ~ 499679.89) | 493.02 (367.01 ~ 658.08) | -0.02(-0.1 to 0.07) |
|  | 50 to 54 | 907090.8 (698966.1 ~ 1120652.78) | 2648.46 (2040.79 ~ 3272) | 2097132.66 (1610013.36 ~ 2614955.99) | 2535.56 (1946.6 ~ 3161.64) | -0.1(-0.16 to -0.04) |
|  | 55 to 59 | 1087607.93 (860059.33 ~ 1372982.25) | 3502.53 (2769.73 ~ 4421.54) | 2463010.56 (1925208.98 ~ 3079377.08) | 3049.47 (2383.62 ~ 3812.6) | -0.43(-0.48 to -0.38) |
|  | 60 to 64 | 1143570.17 (900355.86 ~ 1456777.74) | 4414.99 (3476.01 ~ 5624.19) | 2073738.04 (1602844.5 ~ 2692252.34) | 3473.37 (2684.66 ~ 4509.34) | -0.76(-0.82 to -0.7) |
|  | 65 to 69 | 1219503.05 (975265.38 ~ 1501956.24) | 5857.84 (4684.65 ~ 7214.59) | 2419333.11 (1899844.34 ~ 3062325.69) | 4473.09 (3512.61 ~ 5661.92) | -0.92(-1 to -0.84) |
|  | 70 to 74 | 1078399.25 (851065.41 ~ 1358574.45) | 7140.19 (5634.99 ~ 8995.26) | 2415378.03 (1884529.47 ~ 3081058.3) | 5447.99 (4250.64 ~ 6949.47) | -1.05(-1.15 to -0.95) |
|  | 75 to 79 | 769563.66 (626822.18 ~ 941349.51) | 7594.98 (6186.23 ~ 9290.37) | 1777215.84 (1416226.57 ~ 2199810.83) | 6171.06 (4917.59 ~ 7638.44) | -0.99(-1.12 to -0.86) |
|  | 80 to 84 | 439221.47 (343807.62 ~ 544452.71) | 7674.4 (6007.26 ~ 9513.08) | 1264769.68 (979304.01 ~ 1599986.16) | 6623.06 (5128.2 ~ 8378.45) | -0.82(-0.95 to -0.69) |
|  | 85 to 89 | 185531.9 (146188.45 ~ 225670.45) | 7586.55 (5977.76 ~ 9227.84) | 817155.98 (643263.69 ~ 998661.11) | 6977.16 (5492.41 ~ 8526.91) | -0.69(-0.84 to -0.53) |
|  | 90 to 94 | 42192.88 (32278.58 ~ 52074.22) | 6204.5 (4746.6 ~ 7657.57) | 319636 (242603.02 ~ 400689.69) | 6158.12 (4674 ~ 7719.71) | -0.44(-0.59 to -0.3) |
|  | 95 plus | 7689.46 (5757.96 ~ 9704.06) | 5296.84 (3966.34 ~ 6684.58) | 91731.33 (68234.54 ~ 115766.01) | 5111.63 (3802.3 ~ 6450.94) | -0.36(-0.46 to -0.26) |
|  | All ages | 12653101.21 (11399608.34 ~ 13847406.71) | 1491.33 (1343.59 ~ 1632.1) | 21958170.15 (19613977.55 ~ 24206531.07) | 2005.81 (1791.67 ~ 2211.19) | 0.81(0.78 to 0.84) |
| Male | 0 to 4 | 122204.8 (92644.83 ~ 163469.32) | 137.6 (104.32 ~ 184.07) | 85389.19 (63788.15 ~ 114778.41) | 139.3 (104.06 ~ 187.25) | 0.04(-0.01 to 0.09) |
|  | 10 to 14 | 590908.77 (424888.83 ~ 764468.15) | 718.93 (516.94 ~ 930.09) | 548756.99 (391699.87 ~ 713580.03) | 702.93 (501.75 ~ 914.06) | 0.05(-0.13 to 0.24) |
|  | 15 to 19 | 743033.9 (565888.89 ~ 941235.08) | 802.21 (610.95 ~ 1016.19) | 559278.83 (419293.93 ~ 709796.53) | 767.67 (575.53 ~ 974.27) | -0.03(-0.19 to 0.12) |
|  | 20 to 24 | 706255.27 (547774.67 ~ 904873.97) | 769.01 (596.45 ~ 985.28) | 494189.19 (378252.74 ~ 630642.17) | 730.48 (559.11 ~ 932.18) | -0.16(-0.23 to -0.08) |
|  | 25 to 29 | 583018.12 (453329.5 ~ 732576.2) | 741.96 (576.91 ~ 932.28) | 503223.88 (388476.79 ~ 634925.83) | 695.92 (537.24 ~ 878.06) | -0.2(-0.25 to -0.16) |
|  | 30 to 34 | 491530.12 (376032.98 ~ 644480.24) | 742.67 (568.16 ~ 973.76) | 600892.34 (457539.32 ~ 791255.76) | 686.98 (523.09 ~ 904.62) | -0.21(-0.25 to -0.18) |
|  | 35 to 39 | 537521.45 (420855.78 ~ 680851.54) | 826.83 (647.38 ~ 1047.31) | 692085.37 (536019.23 ~ 870693.75) | 766.03 (593.29 ~ 963.73) | -0.19(-0.22 to -0.17) |
|  | 40 to 44 | 529905.88 (405456.66 ~ 691104.86) | 1042.98 (798.03 ~ 1360.25) | 734728.75 (556328.57 ~ 956829.7) | 977.91 (740.46 ~ 1273.52) | -0.18(-0.21 to -0.16) |
|  | 45 to 49 | 614684.95 (468008.23 ~ 772027.66) | 1532.04 (1166.46 ~ 1924.2) | 1124843.05 (854270.61 ~ 1403577.6) | 1463.69 (1111.61 ~ 1826.39) | -0.15(-0.22 to -0.08) |
|  | 5 to 9 | 359652.87 (263391.2 ~ 478844.02) | 423.59 (310.21 ~ 563.97) | 350166.91 (261135.7 ~ 470627.58) | 416.39 (310.52 ~ 559.63) | 0.04(-0.05 to 0.13) |
|  | 50 to 54 | 787781.31 (603375.04 ~ 977979.76) | 2153.74 (1649.59 ~ 2673.73) | 1679186.99 (1286313.06 ~ 2107268.88) | 1982.83 (1518.91 ~ 2488.32) | -0.27(-0.33 to -0.21) |
|  | 55 to 59 | 969438.46 (763198.69 ~ 1230327.54) | 2981.13 (2346.92 ~ 3783.39) | 1994487.52 (1564585.38 ~ 2492135.05) | 2495.68 (1957.75 ~ 3118.38) | -0.62(-0.66 to -0.57) |
|  | 60 to 64 | 1029773.99 (811307.33 ~ 1312138.84) | 3929.37 (3095.76 ~ 5006.81) | 1728822.2 (1328537.95 ~ 2243291.71) | 2955.24 (2271 ~ 3834.67) | -0.97(-1.01 to -0.92) |
|  | 65 to 69 | 1028057.09 (820647.99 ~ 1270039.44) | 5383.05 (4297.03 ~ 6650.11) | 1970351.04 (1546948.82 ~ 2479942.46) | 3794.73 (2979.29 ~ 4776.16) | -1.18(-1.23 to -1.13) |
|  | 70 to 74 | 829765.85 (649928.02 ~ 1042419.27) | 6638.22 (5199.49 ~ 8339.47) | 1866733.79 (1446392.1 ~ 2395987.53) | 4616.93 (3577.31 ~ 5925.91) | -1.31(-1.37 to -1.25) |
|  | 75 to 79 | 528178 (426689.32 ~ 645508.11) | 7123.27 (5754.54 ~ 8705.64) | 1319002.02 (1051857.89 ~ 1632271.57) | 5344.03 (4261.68 ~ 6613.27) | -1.17(-1.26 to -1.09) |
|  | 80 to 84 | 245277.93 (190746.01 ~ 304898.88) | 7142.13 (5554.24 ~ 8878.2) | 872041.38 (671053.55 ~ 1092138.1) | 5922.77 (4557.69 ~ 7417.63) | -0.9(-0.98 to -0.81) |
|  | 85 to 89 | 83735.75 (65356.52 ~ 102327.19) | 6974.85 (5443.93 ~ 8523.44) | 479728.08 (372538.67 ~ 595863.69) | 6354.15 (4934.39 ~ 7892.41) | -0.6(-0.7 to -0.51) |
|  | 90 to 94 | 14853.42 (11203.11 ~ 18744.78) | 5715.06 (4310.55 ~ 7212.31) | 152133.12 (113347.71 ~ 194352.06) | 5652.19 (4211.2 ~ 7220.75) | -0.22(-0.3 to -0.14) |
|  | 95 plus | 2246.94 (1674.5 ~ 2885.65) | 5085.44 (3789.85 ~ 6531.01) | 33427.06 (24829.08 ~ 42807.18) | 4873.15 (3619.7 ~ 6240.62) | -0.1(-0.19 to 0) |
|  | All ages | 10797824.87 (9760331.95 ~ 11857798.9) | 1226.57 (1108.72 ~ 1346.98) | 17789467.68 (15920345.14 ~ 19644607.82) | 1571.69 (1406.55 ~ 1735.59) | 0.7(0.66 to 0.73) |

**Abbreviations:** CI, Confidence interval; UI, Uncertainty Interval; EAPC, Estimated Annual Percentage Change

**Notes:**Rates for individual age groups are age-specific prevalence rates. The “All ages” rate represents the crude all-age prevalence rate based on the observed population structure and is not age-standardised; therefore, it is not directly comparable with the ASPR reported in Appendix Table 4. EAPCs in this table were estimated from the corresponding age-specific or crude all-age prevalence rates.

| **Appendix Table 7. DALYs of refractive disorders across sex and age subgroups in the Western Pacific Region from 1990 to 2023** | | | | | | |
| --- | --- | --- | --- | --- | --- | --- |
| Sex | Age | 1990 Year | | 2023 Year | | EAPC (95%CI) |
|  |  | Number (95%UI) | Rate (95%UI) | Number (95%UI) | Rate (95%UI) |  |
| Both | 0 to 4 | 9435.75 (6010.42 ~ 14738.45) | 5.55 (3.54 ~ 8.67) | 6386.57 (3987 ~ 10048.81) | 5.47 (3.42 ~ 8.61) | -0.01(-0.06 to 0.04) |
|  | 5 to 9 | 28809.05 (17401.48 ~ 45545.65) | 17.56 (10.61 ~ 27.76) | 26645.18 (15865.36 ~ 41773.37) | 16.65 (9.91 ~ 26.1) | -0.02(-0.1 to 0.06) |
|  | 10 to 14 | 46771.69 (28547.3 ~ 73729.3) | 29.27 (17.87 ~ 46.15) | 40670.66 (24843.21 ~ 63973.11) | 27.36 (16.72 ~ 43.04) | -0.06(-0.21 to 0.1) |
|  | 15 to 19 | 60794.94 (39324 ~ 93031.71) | 33.49 (21.66 ~ 51.24) | 41665.95 (26831.54 ~ 64602.74) | 30.12 (19.4 ~ 46.7) | -0.21(-0.35 to -0.08) |
|  | 20 to 24 | 61841.55 (41265.64 ~ 93286.72) | 34.11 (22.76 ~ 51.45) | 37708.09 (25006.22 ~ 58493.99) | 29.26 (19.41 ~ 45.39) | -0.43(-0.5 to -0.35) |
|  | 25 to 29 | 54305.27 (36172.1 ~ 81302.55) | 35.02 (23.33 ~ 52.43) | 40710.66 (27227.81 ~ 62160.76) | 29.53 (19.75 ~ 45.09) | -0.52(-0.59 to -0.45) |
|  | 30 to 34 | 47255.51 (31757.27 ~ 71861.55) | 36.71 (24.67 ~ 55.82) | 52812.7 (34527.54 ~ 81563.83) | 31.46 (20.57 ~ 48.59) | -0.48(-0.58 to -0.39) |
|  | 35 to 39 | 54457.16 (36798.57 ~ 80169.99) | 42.95 (29.02 ~ 63.23) | 65096.51 (43480.75 ~ 97827.28) | 36.99 (24.71 ~ 55.59) | -0.47(-0.56 to -0.37) |
|  | 40 to 44 | 54263.55 (36339.34 ~ 79268.51) | 55.21 (36.98 ~ 80.66) | 69743.35 (46459 ~ 104531.19) | 47.51 (31.65 ~ 71.21) | -0.44(-0.53 to -0.35) |
|  | 45 to 49 | 62557.23 (43413.35 ~ 91181.52) | 80.51 (55.88 ~ 117.36) | 107619.62 (74335.94 ~ 158697.68) | 70.99 (49.03 ~ 104.68) | -0.35(-0.47 to -0.23) |
|  | 50 to 54 | 79424.84 (53185.19 ~ 114509.62) | 112.14 (75.09 ~ 161.67) | 161204.25 (108026.62 ~ 231361.54) | 96.3 (64.53 ~ 138.21) | -0.41(-0.51 to -0.31) |
|  | 55 to 59 | 98353.47 (65188.01 ~ 139082.1) | 154.71 (102.54 ~ 218.78) | 196627.72 (130722.83 ~ 280661.49) | 122.37 (81.35 ~ 174.66) | -0.73(-0.81 to -0.66) |
|  | 60 to 64 | 106385.13 (70738.69 ~ 151582.12) | 204.16 (135.75 ~ 290.89) | 171794.58 (114085.21 ~ 245087.93) | 145.34 (96.52 ~ 207.34) | -1.06(-1.13 to -0.98) |
|  | 65 to 69 | 112243.47 (74428.63 ~ 156487.46) | 281.2 (186.46 ~ 392.04) | 203140.51 (134206.61 ~ 282636.11) | 191.62 (126.6 ~ 266.61) | -1.22(-1.28 to -1.16) |
|  | 70 to 74 | 97989.27 (64233.31 ~ 135939.47) | 354.99 (232.7 ~ 492.48) | 201577.07 (131773.59 ~ 283602.24) | 237.8 (155.45 ~ 334.56) | -1.38(-1.44 to -1.33) |
|  | 75 to 79 | 67758.84 (46112.29 ~ 92229.05) | 386.15 (262.79 ~ 525.6) | 145604.2 (98815.69 ~ 203111.17) | 272.25 (184.77 ~ 379.78) | -1.34(-1.42 to -1.26) |
|  | All ages | 1093865.23 (780125.62 ~ 1482531.92) | 63.27 (45.13 ~ 85.76) | 1747770.8 (1227539.87 ~ 2319172.6) | 78.5 (55.13 ~ 104.16) | 0.61(0.55 to 0.67) |
|  | 80 to 84 | 35210.53 (23869.43 ~ 48741.39) | 384.5 (260.66 ~ 532.26) | 97974.73 (65458.89 ~ 138414.68) | 289.69 (193.55 ~ 409.27) | -1.16(-1.24 to -1.08) |
|  | 85 to 89 | 13087.36 (8888.91 ~ 17808.38) | 358.94 (243.79 ~ 488.43) | 56906.16 (38194 ~ 78047.51) | 295.44 (198.29 ~ 405.2) | -0.96(-1.05 to -0.87) |
|  | 90 to 94 | 2508.24 (1716.85 ~ 3333.83) | 266.85 (182.66 ~ 354.69) | 19144.33 (12807.25 ~ 25671.14) | 242.88 (162.49 ~ 325.69) | -0.66(-0.77 to -0.54) |
|  | 95 plus | 412.38 (277.98 ~ 556.86) | 217.78 (146.8 ~ 294.08) | 4737.97 (3181.39 ~ 6575.96) | 191.01 (128.26 ~ 265.11) | -0.61(-0.71 to -0.52) |
| Female | 0 to 4 | 4940.92 (3133.99 ~ 7759.28) | 6.09 (3.86 ~ 9.56) | 3249.92 (1991.85 ~ 5142.88) | 5.87 (3.6 ~ 9.29) | -0.07(-0.11 to -0.02) |
|  | 5 to 9 | 14909.28 (9219.57 ~ 23485.72) | 18.83 (11.65 ~ 29.67) | 13322.78 (8014.4 ~ 21670.39) | 17.55 (10.55 ~ 28.54) | -0.08(-0.15 to 0) |
|  | 10 to 14 | 23652.64 (14331.31 ~ 37382.92) | 30.49 (18.47 ~ 48.19) | 19988.67 (12088.6 ~ 31543.45) | 28.33 (17.13 ~ 44.71) | -0.11(-0.26 to 0.05) |
|  | 15 to 19 | 29883.59 (19121.46 ~ 46203.73) | 33.61 (21.5 ~ 51.96) | 19801.9 (12602.06 ~ 30892.97) | 30.24 (19.25 ~ 47.18) | -0.26(-0.41 to -0.1) |
|  | 20 to 24 | 29645 (19778.77 ~ 45391.65) | 33.14 (22.11 ~ 50.74) | 17348.33 (11355.76 ~ 27358.47) | 28.34 (18.55 ~ 44.7) | -0.46(-0.58 to -0.34) |
|  | 25 to 29 | 26358.76 (17416.09 ~ 39227.17) | 34.46 (22.77 ~ 51.29) | 18956.71 (12648.17 ~ 28931.63) | 28.91 (19.29 ~ 44.13) | -0.53(-0.65 to -0.41) |
|  | 30 to 34 | 23623.45 (15735.33 ~ 36270.95) | 37.76 (25.15 ~ 57.98) | 26091.78 (16967.61 ~ 40854.85) | 32.46 (21.11 ~ 50.82) | -0.47(-0.61 to -0.34) |
|  | 35 to 39 | 28851.78 (19237.48 ~ 42397.38) | 46.7 (31.14 ~ 68.62) | 34470.44 (23113.83 ~ 51299.63) | 40.25 (26.99 ~ 59.9) | -0.47(-0.59 to -0.34) |
|  | 40 to 44 | 29165.58 (19702.25 ~ 42018.79) | 61.44 (41.5 ~ 88.51) | 38007.85 (25493.94 ~ 57126.51) | 53.04 (35.58 ~ 79.72) | -0.43(-0.54 to -0.31) |
|  | 45 to 49 | 33727.28 (23344.88 ~ 49125.16) | 89.76 (62.13 ~ 130.74) | 59884.9 (40821 ~ 87696.77) | 80.11 (54.61 ~ 117.32) | -0.31(-0.42 to -0.19) |
|  | 50 to 54 | 42407.79 (28662.88 ~ 61144.56) | 123.82 (83.69 ~ 178.53) | 89499.92 (60346.42 ~ 129504.32) | 108.21 (72.96 ~ 156.58) | -0.34(-0.43 to -0.24) |
|  | 55 to 59 | 52115.52 (34592.32 ~ 74300.94) | 167.83 (111.4 ~ 239.28) | 109221.19 (72989.12 ~ 155739.14) | 135.23 (90.37 ~ 192.82) | -0.65(-0.72 to -0.57) |
|  | 60 to 64 | 56316.98 (37395.19 ~ 79817.99) | 217.42 (144.37 ~ 308.15) | 94433.82 (63215.33 ~ 134702.3) | 158.17 (105.88 ~ 225.62) | -0.96(-1.03 to -0.9) |
|  | 65 to 69 | 61423.87 (40634.2 ~ 85584.76) | 295.05 (195.18 ~ 411.1) | 112747.54 (74299.72 ~ 156989.16) | 208.46 (137.37 ~ 290.26) | -1.1(-1.15 to -1.06) |
|  | 70 to 74 | 56128.73 (36779.82 ~ 77415.84) | 371.63 (243.52 ~ 512.58) | 114671.56 (74600.44 ~ 160067.81) | 258.65 (168.26 ~ 361.04) | -1.26(-1.31 to -1.21) |
|  | 75 to 79 | 40801.99 (27693.13 ~ 55862.76) | 402.68 (273.31 ~ 551.32) | 84474.07 (57615.51 ~ 117030.23) | 293.32 (200.06 ~ 406.37) | -1.25(-1.33 to -1.17) |
|  | All ages | 588317.68 (417991.62 ~ 796561.78) | 69.34 (49.27 ~ 93.89) | 967520.79 (681303.21 ~ 1279464.25) | 88.38 (62.23 ~ 116.87) | 0.67(0.62 to 0.73) |
|  | 80 to 84 | 22977.04 (15621.79 ~ 31806.08) | 401.47 (272.96 ~ 555.74) | 58583.74 (38919.85 ~ 81982.88) | 306.78 (203.81 ~ 429.31) | -1.12(-1.21 to -1.04) |
|  | 85 to 89 | 9180.93 (6323.03 ~ 12559.55) | 375.42 (258.55 ~ 513.57) | 36215.37 (24305.68 ~ 49623.13) | 309.22 (207.53 ~ 423.7) | -0.99(-1.1 to -0.87) |
|  | 90 to 94 | 1883.3 (1296.02 ~ 2495.21) | 276.94 (190.58 ~ 366.92) | 13067.18 (8750 ~ 17466.71) | 251.75 (168.58 ~ 336.51) | -0.73(-0.86 to -0.59) |
|  | 95 plus | 323.25 (218.04 ~ 434.71) | 222.67 (150.19 ~ 299.45) | 3483.13 (2357.18 ~ 4816.52) | 194.09 (131.35 ~ 268.4) | -0.7(-0.8 to -0.59) |
| Male | 0 to 4 | 4494.84 (2792.21 ~ 6983.86) | 5.06 (3.14 ~ 7.86) | 3136.65 (1910.91 ~ 4942.87) | 5.12 (3.12 ~ 8.06) | 0.05(-0.01 to 0.11) |
|  | 5 to 9 | 13899.77 (8380.61 ~ 21970.02) | 16.37 (9.87 ~ 25.88) | 13322.4 (7913.37 ~ 20405.67) | 15.84 (9.41 ~ 24.26) | 0.04(-0.04 to 0.13) |
|  | 10 to 14 | 23119.05 (14159.93 ~ 36558.18) | 28.13 (17.23 ~ 44.48) | 20681.99 (12553.92 ~ 32125.52) | 26.49 (16.08 ~ 41.15) | 0.01(-0.15 to 0.17) |
|  | 15 to 19 | 30911.34 (19932.66 ~ 47231.94) | 33.37 (21.52 ~ 50.99) | 21864.05 (14225.26 ~ 33843.8) | 30.01 (19.53 ~ 46.45) | -0.17(-0.29 to -0.05) |
|  | 20 to 24 | 32196.55 (21208.28 ~ 48097.35) | 35.06 (23.09 ~ 52.37) | 20359.76 (13476.68 ~ 31827.11) | 30.09 (19.92 ~ 47.04) | -0.4(-0.45 to -0.36) |
|  | 25 to 29 | 27946.52 (18767.44 ~ 42556.9) | 35.57 (23.88 ~ 54.16) | 21753.95 (14323.23 ~ 33356.61) | 30.08 (19.81 ~ 46.13) | -0.52(-0.54 to -0.49) |
|  | 30 to 34 | 23632.05 (15905.5 ~ 35527.06) | 35.71 (24.03 ~ 53.68) | 26720.93 (17534.18 ~ 40866.01) | 30.55 (20.05 ~ 46.72) | -0.49(-0.54 to -0.44) |
|  | 35 to 39 | 25605.38 (17631.68 ~ 38068.41) | 39.39 (27.12 ~ 58.56) | 30626.07 (20514.23 ~ 46121.42) | 33.9 (22.71 ~ 51.05) | -0.47(-0.53 to -0.42) |
|  | 40 to 44 | 25097.97 (16684.48 ~ 37195.1) | 49.4 (32.84 ~ 73.21) | 31735.5 (20965.05 ~ 47473.48) | 42.24 (27.9 ~ 63.19) | -0.47(-0.54 to -0.41) |
|  | 45 to 49 | 28829.95 (19928.67 ~ 41834.47) | 71.86 (49.67 ~ 104.27) | 47734.72 (33067.05 ~ 71096.01) | 62.11 (43.03 ~ 92.51) | -0.42(-0.54 to -0.3) |
|  | 50 to 54 | 37017.05 (24707.33 ~ 53324.81) | 101.2 (67.55 ~ 145.79) | 71704.32 (47448.48 ~ 103061.71) | 84.67 (56.03 ~ 121.7) | -0.52(-0.62 to -0.41) |
|  | 55 to 59 | 46237.95 (30468.89 ~ 65352.58) | 142.19 (93.7 ~ 200.97) | 87406.52 (58114.74 ~ 124835.33) | 109.37 (72.72 ~ 156.21) | -0.85(-0.93 to -0.77) |
|  | 60 to 64 | 50068.14 (33409.53 ~ 70899.38) | 191.05 (127.48 ~ 270.54) | 77360.76 (51126.03 ~ 108852.37) | 132.24 (87.39 ~ 186.07) | -1.18(-1.27 to -1.09) |
|  | 65 to 69 | 50819.61 (33698.62 ~ 70702.85) | 266.1 (176.45 ~ 370.21) | 90392.96 (60020.53 ~ 126009.41) | 174.09 (115.59 ~ 242.68) | -1.36(-1.44 to -1.28) |
|  | 70 to 74 | 41860.54 (27453.49 ~ 58068.78) | 334.89 (219.63 ~ 464.56) | 86905.51 (56377.61 ~ 123007.8) | 214.94 (139.44 ~ 304.23) | -1.52(-1.59 to -1.44) |
|  | 75 to 79 | 26956.85 (18419.16 ~ 37092) | 363.55 (248.41 ~ 500.24) | 61130.14 (41161.31 ~ 85788.55) | 247.67 (166.77 ~ 347.58) | -1.42(-1.52 to -1.32) |
|  | All ages | 505547.56 (362134 ~ 685970.14) | 57.43 (41.14 ~ 77.92) | 780250.01 (545767.72 ~ 1041634.32) | 68.93 (48.22 ~ 92.03) | 0.52(0.45 to 0.6) |
|  | 80 to 84 | 12233.49 (8247.64 ~ 16972.11) | 356.22 (240.16 ~ 494.2) | 39390.99 (25990.11 ~ 56528.76) | 267.54 (176.52 ~ 383.93) | -1.17(-1.27 to -1.06) |
|  | 85 to 89 | 3906.43 (2637.93 ~ 5317.47) | 325.39 (219.73 ~ 442.92) | 20690.8 (13929.14 ~ 28494.47) | 274.06 (184.5 ~ 377.42) | -0.83(-0.91 to -0.74) |
|  | 90 to 94 | 624.94 (421.59 ~ 850.04) | 240.45 (162.21 ~ 327.06) | 6077.15 (4066.95 ~ 8252.68) | 225.78 (151.1 ~ 306.61) | -0.39(-0.46 to -0.32) |
|  | 95 plus | 89.13 (59.9 ~ 123.16) | 201.73 (135.58 ~ 278.73) | 1254.84 (824.17 ~ 1747.71) | 182.94 (120.15 ~ 254.79) | -0.29(-0.38 to -0.2) |

**Abbreviations:** CI, Confidence interval; UI, Uncertainty Interval; EAPC, Estimated Annual Percentage Change

**Notes:**Rates for individual age groups are age-specific DALY rates. The “All ages” rate represents the crude all-age DALY rate based on the observed population structure and is not age-standardised; therefore, it is not directly comparable with the ASDR reported in Appendix Table 5. EAPCs were estimated from the corresponding age-specific or crude all-age DALY rates.

| **Appendix Table 8. APC of Joinpoint analysis** | | | | | | |
| --- | --- | --- | --- | --- | --- | --- |
| Measure | Location | Sex | Cause | Year Range | APC (95%CI) | P Value |
| Prevalence | Western Pacific Region | Both | Refraction disorders | 1990 to 1996 year | -0.13(-0.2 to -0.03) | 0.016 |
| Prevalence | Western Pacific Region | Both | Refraction disorders | 1996 to 1999 year | -1.06(-1.22 to -0.8) | 0.001 |
| Prevalence | Western Pacific Region | Both | Refraction disorders | 1999 to 2004 year | -0.29(-0.39 to -0.04) | 0.022 |
| Prevalence | Western Pacific Region | Both | Refraction disorders | 2004 to 2016 year | -0.52(-0.56 to -0.49) | <0.001 |
| Prevalence | Western Pacific Region | Both | Refraction disorders | 2016 to 2019 year | -1.65(-1.79 to -1.37) | <0.001 |
| Prevalence | Western Pacific Region | Both | Refraction disorders | 2019 to 2023 year | 0.42(0.27 to 0.56) | <0.001 |
| Prevalence | Western Pacific Region | Female | Refraction disorders | 1990 to 1996 year | -0.09(-0.16 to 0.01) | 0.072 |
| Prevalence | Western Pacific Region | Female | Refraction disorders | 1996 to 1999 year | -1.08(-1.24 to -0.81) | <0.001 |
| Prevalence | Western Pacific Region | Female | Refraction disorders | 1999 to 2005 year | -0.24(-0.31 to 0.04) | 0.066 |
| Prevalence | Western Pacific Region | Female | Refraction disorders | 2005 to 2016 year | -0.48(-0.52 to -0.44) | <0.001 |
| Prevalence | Western Pacific Region | Female | Refraction disorders | 2016 to 2019 year | -1.6(-1.74 to -1.32) | <0.001 |
| Prevalence | Western Pacific Region | Female | Refraction disorders | 2019 to 2023 year | 0.43(0.28 to 0.57) | <0.001 |
| Prevalence | Western Pacific Region | Male | Refraction disorders | 1990 to 1995 year | -0.21(-0.36 to 0.06) | 0.086 |
| Prevalence | Western Pacific Region | Male | Refraction disorders | 1995 to 2016 year | -0.56(-0.59 to -0.54) | <0.001 |
| Prevalence | Western Pacific Region | Male | Refraction disorders | 2016 to 2019 year | -1.75(-1.93 to -1.38) | <0.001 |
| Prevalence | Western Pacific Region | Male | Refraction disorders | 2019 to 2023 year | 0.39(0.18 to 0.61) | <0.001 |
| DALYs (Disability-Adjusted Life Years) | Western Pacific Region | Both | Refraction disorders | 1990 to 1993 year | -0.96(-1.34 to -0.72) | <0.001 |
| DALYs (Disability-Adjusted Life Years) | Western Pacific Region | Both | Refraction disorders | 1993 to 2001 year | -0.22(-0.29 to -0.14) | 0.001 |
| DALYs (Disability-Adjusted Life Years) | Western Pacific Region | Both | Refraction disorders | 2001 to 2010 year | -0.95(-1.04 to -0.9) | <0.001 |
| DALYs (Disability-Adjusted Life Years) | Western Pacific Region | Both | Refraction disorders | 2010 to 2015 year | -0.57(-0.68 to -0.39) | <0.001 |
| DALYs (Disability-Adjusted Life Years) | Western Pacific Region | Both | Refraction disorders | 2015 to 2020 year | -1.9(-2 to -1.8) | <0.001 |
| DALYs (Disability-Adjusted Life Years) | Western Pacific Region | Both | Refraction disorders | 2020 to 2023 year | 0.74(0.56 to 0.92) | <0.001 |
| DALYs (Disability-Adjusted Life Years) | Western Pacific Region | Female | Refraction disorders | 1990 to 1993 year | -0.83(-1.17 to -0.59) | <0.001 |
| DALYs (Disability-Adjusted Life Years) | Western Pacific Region | Female | Refraction disorders | 1993 to 2002 year | -0.3(-0.36 to -0.22) | 0.001 |
| DALYs (Disability-Adjusted Life Years) | Western Pacific Region | Female | Refraction disorders | 2002 to 2010 year | -0.9(-1 to -0.83) | <0.001 |
| DALYs (Disability-Adjusted Life Years) | Western Pacific Region | Female | Refraction disorders | 2010 to 2015 year | -0.46(-0.57 to -0.31) | <0.001 |
| DALYs (Disability-Adjusted Life Years) | Western Pacific Region | Female | Refraction disorders | 2015 to 2020 year | -1.89(-1.99 to -1.81) | <0.001 |
| DALYs (Disability-Adjusted Life Years) | Western Pacific Region | Female | Refraction disorders | 2020 to 2023 year | 0.78(0.61 to 0.94) | <0.001 |
| DALYs (Disability-Adjusted Life Years) | Western Pacific Region | Male | Refraction disorders | 1990 to 1994 year | -0.86(-1.12 to -0.69) | <0.001 |
| DALYs (Disability-Adjusted Life Years) | Western Pacific Region | Male | Refraction disorders | 1994 to 2001 year | -0.07(-0.15 to 0.02) | 0.1 |
| DALYs (Disability-Adjusted Life Years) | Western Pacific Region | Male | Refraction disorders | 2001 to 2010 year | -1.1(-1.17 to -1.04) | <0.001 |
| DALYs (Disability-Adjusted Life Years) | Western Pacific Region | Male | Refraction disorders | 2010 to 2015 year | -0.64(-0.77 to -0.45) | <0.001 |
| DALYs (Disability-Adjusted Life Years) | Western Pacific Region | Male | Refraction disorders | 2015 to 2020 year | -1.94(-2.06 to -1.84) | <0.001 |
| DALYs (Disability-Adjusted Life Years) | Western Pacific Region | Male | Refraction disorders | 2020 to 2023 year | 0.66(0.47 to 0.84) | <0.001 |

**Abbreviations:** CI, Confidence interval; APC, annual percent change

**Notes:** APC of Joinpoint analysis

| **Appendix Table 9. AAPC of Joinpoint analysis** | | | | | | |
| --- | --- | --- | --- | --- | --- | --- |
| Measure | Location | Sex | Cause | Year Range | AAPC (95%CI) | P value |
| DALYs (Disability-Adjusted Life Years) | Western Pacific Region | Both | Refraction disorders | Last 5 year (2019 to 2023 year) | 0.07(-0.05 to 0.19) | 0.21 |
| DALYs (Disability-Adjusted Life Years) | Western Pacific Region | Both | Refraction disorders | Last 10 year (2014 to 2023 year) | -0.88(-0.93 to -0.84) | <0.001 |
| DALYs (Disability-Adjusted Life Years) | Western Pacific Region | Both | Refraction disorders | 1990 to 1999 year | -0.47(-0.52 to -0.42) | <0.001 |
| DALYs (Disability-Adjusted Life Years) | Western Pacific Region | Both | Refraction disorders | 2000 to 2009 year | -0.87(-0.92 to -0.83) | <0.001 |
| DALYs (Disability-Adjusted Life Years) | Western Pacific Region | Both | Refraction disorders | 2010 to 2019 year | -1.16(-1.21 to -1.13) | <0.001 |
| DALYs (Disability-Adjusted Life Years) | Western Pacific Region | Both | Refraction disorders | 1990 to 2023 year | -0.71(-0.73 to -0.69) | <0.001 |
| DALYs (Disability-Adjusted Life Years) | Western Pacific Region | Female | Refraction disorders | Last 5 year (2019 to 2023 year) | 0.1(-0.02 to 0.21) | 0.081 |
| DALYs (Disability-Adjusted Life Years) | Western Pacific Region | Female | Refraction disorders | Last 10 year (2014 to 2023 year) | -0.85(-0.9 to -0.81) | <0.001 |
| DALYs (Disability-Adjusted Life Years) | Western Pacific Region | Female | Refraction disorders | 1990 to 1999 year | -0.48(-0.52 to -0.43) | <0.001 |
| DALYs (Disability-Adjusted Life Years) | Western Pacific Region | Female | Refraction disorders | 2000 to 2009 year | -0.77(-0.81 to -0.73) | <0.001 |
| DALYs (Disability-Adjusted Life Years) | Western Pacific Region | Female | Refraction disorders | 2010 to 2019 year | -1.1(-1.15 to -1.07) | <0.001 |
| DALYs (Disability-Adjusted Life Years) | Western Pacific Region | Female | Refraction disorders | 1990 to 2023 year | -0.66(-0.68 to -0.65) | <0.001 |
| DALYs (Disability-Adjusted Life Years) | Western Pacific Region | Male | Refraction disorders | Last 5 year (2019 to 2023 year) | 0(-0.13 to 0.12) | 0.933 |
| DALYs (Disability-Adjusted Life Years) | Western Pacific Region | Male | Refraction disorders | Last 10 year (2014 to 2023 year) | -0.94(-0.99 to -0.89) | <0.001 |
| DALYs (Disability-Adjusted Life Years) | Western Pacific Region | Male | Refraction disorders | 1990 to 1999 year | -0.42(-0.47 to -0.37) | <0.001 |
| DALYs (Disability-Adjusted Life Years) | Western Pacific Region | Male | Refraction disorders | 2000 to 2009 year | -0.98(-1.03 to -0.94) | <0.001 |
| DALYs (Disability-Adjusted Life Years) | Western Pacific Region | Male | Refraction disorders | 2010 to 2019 year | -1.22(-1.27 to -1.19) | <0.001 |
| DALYs (Disability-Adjusted Life Years) | Western Pacific Region | Male | Refraction disorders | 1990 to 2023 year | -0.75(-0.77 to -0.74) | <0.001 |
| Prevalence | Western Pacific Region | Both | Refraction disorders | Last 5 year (2019 to 2023 year) | 0.42(0.27 to 0.55) | <0.001 |
| Prevalence | Western Pacific Region | Both | Refraction disorders | Last 10 year (2014 to 2023 year) | -0.48(-0.53 to -0.45) | <0.001 |
| Prevalence | Western Pacific Region | Both | Refraction disorders | 1990 to 1999 year | -0.44(-0.49 to -0.39) | <0.001 |
| Prevalence | Western Pacific Region | Both | Refraction disorders | 2000 to 2009 year | -0.42(-0.44 to -0.36) | <0.001 |
| Prevalence | Western Pacific Region | Both | Refraction disorders | 2010 to 2019 year | -0.9(-0.94 to -0.86) | <0.001 |
| Prevalence | Western Pacific Region | Both | Refraction disorders | 1990 to 2023 year | -0.45(-0.47 to -0.44) | <0.001 |
| Prevalence | Western Pacific Region | Female | Refraction disorders | Last 5 year (2019 to 2023 year) | 0.43(0.28 to 0.56) | <0.001 |
| Prevalence | Western Pacific Region | Female | Refraction disorders | Last 10 year (2014 to 2023 year) | -0.45(-0.5 to -0.42) | <0.001 |
| Prevalence | Western Pacific Region | Female | Refraction disorders | 1990 to 1999 year | -0.42(-0.47 to -0.36) | <0.001 |
| Prevalence | Western Pacific Region | Female | Refraction disorders | 2000 to 2009 year | -0.34(-0.37 to -0.29) | <0.001 |
| Prevalence | Western Pacific Region | Female | Refraction disorders | 2010 to 2019 year | -0.85(-0.9 to -0.82) | <0.001 |
| Prevalence | Western Pacific Region | Female | Refraction disorders | 1990 to 2023 year | -0.41(-0.43 to -0.4) | <0.001 |
| Prevalence | Western Pacific Region | Male | Refraction disorders | Last 5 year (2019 to 2023 year) | 0.39(0.18 to 0.58) | <0.001 |
| Prevalence | Western Pacific Region | Male | Refraction disorders | Last 10 year (2014 to 2023 year) | -0.54(-0.61 to -0.49) | <0.001 |
| Prevalence | Western Pacific Region | Male | Refraction disorders | 1990 to 1999 year | -0.37(-0.41 to -0.3) | <0.001 |
| Prevalence | Western Pacific Region | Male | Refraction disorders | 2000 to 2009 year | -0.56(-0.59 to -0.54) | <0.001 |
| Prevalence | Western Pacific Region | Male | Refraction disorders | 2010 to 2019 year | -0.96(-1.02 to -0.9) | <0.001 |
| Prevalence | Western Pacific Region | Male | Refraction disorders | 1990 to 2023 year | -0.5(-0.52 to -0.48) | <0.001 |

**Abbreviations:** CI, Confidence interval; AAPC, average annual percent change

**Notes:** AAPC of Joinpoint analysis

| **Appendix Table 10. Decomposition analysis results of refractive disorders in the Western Pacific Region** | | | | | |
| --- | --- | --- | --- | --- | --- |
| Location | measure | Overall difference | Population ageing | Population growth | Epidemiological change |
| American Samoa | Prevalence | 244.86(104.01%) | 11.03(4.68%) | 257.61(109.43%) | -23.78(-10.1%) |
| Australia | Prevalence | 109315(149.48%) | 12764.33(17.45%) | 95532.15(130.63%) | 1018.52(1.39%) |
| Brunei Darussalam | Prevalence | 1258.39(292.05%) | -0.8(-0.19%) | 1347.31(312.69%) | -88.13(-20.45%) |
| Cambodia | Prevalence | 102025.63(156.81%) | 4277.32(6.57%) | 122211.25(187.84%) | -24462.94(-37.6%) |
| China | Prevalence | 13419106.93(117.22%) | 1769591.86(15.46%) | 16826290.67(146.98%) | -5176775.6(-45.22%) |
| Cook Islands | Prevalence | 63.39(49.45%) | 15.86(12.38%) | 60.13(46.91%) | -12.6(-9.83%) |
| Fiji | Prevalence | 5483.66(111.93%) | 553.37(11.29%) | 5433.35(110.9%) | -503.05(-10.27%) |
| Guam | Prevalence | 1291.31(164.43%) | 179.78(22.89%) | 1199.12(152.69%) | -87.58(-11.15%) |
| Japan | Prevalence | 440023.27(79.95%) | 149322.11(27.13%) | 317303.41(57.65%) | -26602.25(-4.83%) |
| Kiribati | Prevalence | 408.7(107.94%) | -1.98(-0.52%) | 438.9(115.92%) | -28.22(-7.45%) |
| Lao People's Democratic Republic | Prevalence | 19567.34(135.34%) | -628.3(-4.35%) | 20910.4(144.63%) | -714.75(-4.94%) |
| Malaysia | Prevalence | 134882.21(203.18%) | 4638.57(6.99%) | 150170.31(226.21%) | -19926.68(-30.02%) |
| Marshall Islands | Prevalence | 96.1(58.77%) | -17.43(-10.66%) | 131.39(80.35%) | -17.86(-10.92%) |
| Micronesia (Federated States of) | Prevalence | 323.8(68.75%) | -28.53(-6.06%) | 394.87(83.84%) | -42.54(-9.03%) |
| Mongolia | Prevalence | 13826.76(141.66%) | -1009.4(-10.34%) | 15768.01(161.55%) | -931.86(-9.55%) |
| Nauru | Prevalence | 20.78(60.05%) | 2.15(6.21%) | 20.74(59.92%) | -2.11(-6.09%) |
| New Zealand | Prevalence | 17888.14(112.05%) | 1371.06(8.59%) | 17354.1(108.71%) | -837.01(-5.24%) |
| Niue | Prevalence | 0.02(0.08%) | 0.14(0.59%) | 1.61(6.92%) | -1.73(-7.43%) |
| Northern Mariana Islands | Prevalence | 338.25(189.68%) | 46.82(26.25%) | 307.16(172.24%) | -15.72(-8.81%) |
| Palau | Prevalence | 130.17(134.02%) | 2.34(2.4%) | 138.05(142.14%) | -10.22(-10.52%) |
| Papua New Guinea | Prevalence | 47437.25(200.4%) | -806.45(-3.41%) | 51143.02(216.06%) | -2899.32(-12.25%) |
| Philippines | Prevalence | 556901.94(170.92%) | 16169.23(4.96%) | 597564.77(183.4%) | -56832.06(-17.44%) |
| Republic of Korea | Prevalence | 341440.49(197.42%) | 45884.75(26.53%) | 326879.82(189%) | -31324.07(-18.11%) |
| Samoa | Prevalence | 748.06(78.18%) | -7.47(-0.78%) | 848.01(88.62%) | -92.48(-9.66%) |
| Singapore | Prevalence | 27510.85(278.9%) | 2080.08(21.09%) | 26996.79(273.69%) | -1566.02(-15.88%) |
| Solomon Islands | Prevalence | 3102.95(195.99%) | -167.69(-10.59%) | 3462.92(218.72%) | -192.28(-12.14%) |
| Tokelau | Prevalence | 2.07(13.54%) | -0.53(-3.47%) | 3.97(25.98%) | -1.37(-8.97%) |
| Tonga | Prevalence | 179.6(36.16%) | 17.85(3.59%) | 227.93(45.89%) | -66.18(-13.32%) |
| Tuvalu | Prevalence | 20.77(29.06%) | 2.73(3.81%) | 24.55(34.33%) | -6.5(-9.09%) |
| Vanuatu | Prevalence | 997.62(191.79%) | -21.26(-4.09%) | 1057.3(203.27%) | -38.42(-7.39%) |
| Viet Nam | Prevalence | 421090.65(169.96%) | -9734.04(-3.93%) | 460034.4(185.67%) | -29209.71(-11.79%) |
| American Samoa | DALYs | 7.02(99.37%) | 0.31(4.46%) | 7.64(108.22%) | -0.94(-13.3%) |
| Australia | DALYs | 3127.23(146.29%) | 332.81(15.57%) | 2774.24(129.77%) | 20.17(0.94%) |
| Brunei Darussalam | DALYs | 37.9(287.36%) | -0.03(-0.21%) | 41(310.9%) | -3.08(-23.33%) |
| Cambodia | DALYs | 3932.52(127.94%) | 244.96(7.97%) | 5486.66(178.5%) | -1799.1(-58.53%) |
| China | DALYs | 578867.11(98.35%) | 90077.85(15.3%) | 832316.11(141.41%) | -343526.86(-58.37%) |
| Cook Islands | DALYs | 1.76(45.75%) | 0.44(11.45%) | 1.78(46.32%) | -0.46(-12.02%) |
| Fiji | DALYs | 156.49(108.15%) | 15.31(10.58%) | 159.06(109.92%) | -17.88(-12.36%) |
| Guam | DALYs | 37.83(159.54%) | 5.06(21.33%) | 35.86(151.24%) | -3.09(-13.03%) |
| Japan | DALYs | 12510.43(75.48%) | 3928.46(23.7%) | 9426.27(56.87%) | -844.29(-5.09%) |
| Kiribati | DALYs | 12.04(105.45%) | -0.05(-0.43%) | 13.15(115.24%) | -1.07(-9.36%) |
| Lao People's Democratic Republic | DALYs | 601.44(122.21%) | -20.94(-4.25%) | 692.15(140.64%) | -69.78(-14.18%) |
| Malaysia | DALYs | 4951.76(176.07%) | 234.69(8.34%) | 6100.4(216.91%) | -1383.32(-49.19%) |
| Marshall Islands | DALYs | 2.77(56.22%) | -0.48(-9.84%) | 3.93(79.74%) | -0.67(-13.69%) |
| Micronesia (Federated States of) | DALYs | 9.22(65.01%) | -0.79(-5.57%) | 11.76(82.96%) | -1.75(-12.37%) |
| Mongolia | DALYs | 430.66(143.63%) | -27.64(-9.22%) | 486.3(162.18%) | -28.01(-9.34%) |
| Nauru | DALYs | 0.6(57.15%) | 0.06(5.79%) | 0.62(59.39%) | -0.08(-8.03%) |
| New Zealand | DALYs | 513.64(110.66%) | 34.85(7.51%) | 502.93(108.35%) | -24.14(-5.2%) |
| Niue | DALYs | -0.01(-1.36%) | 0.01(0.78%) | 0.05(6.88%) | -0.06(-9.02%) |
| Northern Mariana Islands | DALYs | 9.94(184.19%) | 1.34(24.79%) | 9.21(170.54%) | -0.6(-11.14%) |
| Palau | DALYs | 3.79(130.44%) | 0.07(2.46%) | 4.1(141.09%) | -0.38(-13.1%) |
| Papua New Guinea | DALYs | 1428.22(197.32%) | -24.49(-3.38%) | 1556.03(214.98%) | -103.32(-14.27%) |
| Philippines | DALYs | 18436.91(158.08%) | 556.63(4.77%) | 20905.07(179.24%) | -3024.79(-25.93%) |
| Republic of Korea | DALYs | 9993.31(191.58%) | 1209.01(23.18%) | 9756.05(187.03%) | -971.75(-18.63%) |
| Samoa | DALYs | 21.74(75.6%) | -0.22(-0.75%) | 25.31(88%) | -3.35(-11.65%) |
| Singapore | DALYs | 825.28(273.11%) | 56.12(18.57%) | 820.52(271.54%) | -51.37(-17%) |
| Solomon Islands | DALYs | 92.57(192.14%) | -4.7(-9.76%) | 104.73(217.38%) | -7.46(-15.48%) |
| Tokelau | DALYs | 0.05(11.4%) | -0.02(-3.44%) | 0.12(25.75%) | -0.05(-10.9%) |
| Tonga | DALYs | 4.75(30.51%) | 0.5(3.2%) | 7(44.98%) | -2.75(-17.66%) |
| Tuvalu | DALYs | 0.58(26.6%) | 0.08(3.62%) | 0.74(34.02%) | -0.24(-11.03%) |
| Vanuatu | DALYs | 29.67(189.59%) | -0.6(-3.81%) | 31.7(202.53%) | -1.43(-9.13%) |
| Viet Nam | DALYs | 16141.84(128.21%) | -608.28(-4.83%) | 21615.5(171.69%) | -4865.38(-38.64%) |

**Abbreviations:** CI, Confidence interval; UI, Uncertainty Interval; EAPC, Estimated Annual Percentage Change

**Notes:**Absolute changes in burden were decomposed into contributions from population ageing, population growth, and epidemiological change using the Shapley decomposition framework. Values outside parentheses represent the estimated absolute contribution of each component. Values in parentheses represent the contribution of each component relative to the corresponding burden in 1990, calculated as the component contribution divided by the 1990 baseline burden ×100%. The overall percentage change represents the observed net change relative to the 1990 baseline. Component percentages therefore sum algebraically to the overall percentage change from baseline rather than to 100%. Positive values indicate increasing effects, whereas negative values indicate offsetting effects.

| **Appendix Table 11. Sex-stratified decomposition of changes in refractive disorder burden across 31 Western Pacific countries and territories, 1990–2023** | | | | | | |
| --- | --- | --- | --- | --- | --- | --- |
| **Location** | **Sex** | **Measure** | **Overall difference** | **Population ageing** | **Population growth** | **Epidemiological change** |
| Solomon Islands | Male | Prevalence | 2466.27 (156.92%) | 261.12 (16.61%) | 2369.42 (150.75%) | -164.27 (-10.45%) |
| Solomon Islands | Male | DALYs | 78.33 (150.68%) | 8.42 (16.2%) | 77.44 (148.97%) | -7.53 (-14.48%) |
| Solomon Islands | Female | Prevalence | 2740.62 (196.24%) | 534.89 (38.3%) | 2337.44 (167.37%) | -131.71 (-9.43%) |
| Solomon Islands | Female | DALYs | 86.24 (186.51%) | 16.23 (35.11%) | 76.05 (164.48%) | -6.05 (-13.08%) |
| Tuvalu | Male | Prevalence | 15.59 (32.93%) | 6.96 (14.7%) | 12.72 (26.87%) | -4.09 (-8.64%) |
| Tuvalu | Male | DALYs | 0.44 (28.81%) | 0.21 (13.8%) | 0.41 (26.45%) | -0.18 (-11.44%) |
| Tuvalu | Female | Prevalence | 8.75 (14.24%) | 7.04 (11.44%) | 6.02 (9.8%) | -4.31 (-7.01%) |
| Tuvalu | Female | DALYs | 0.21 (10.59%) | 0.21 (10.54%) | 0.19 (9.64%) | -0.19 (-9.6%) |
| Tokelau | Male | Prevalence | 0.81 (8.08%) | 1.53 (15.14%) | 0.17 (1.72%) | -0.89 (-8.78%) |
| Tokelau | Male | DALYs | 0.01 (4.63%) | 0.05 (14.31%) | 0.01 (1.69%) | -0.04 (-11.37%) |
| Tokelau | Female | Prevalence | 1.07 (9.5%) | 2.33 (20.77%) | -0.44 (-3.91%) | -0.83 (-7.37%) |
| Tokelau | Female | DALYs | 0.02 (6.15%) | 0.07 (19.63%) | -0.01 (-3.84%) | -0.03 (-9.64%) |
| Tonga | Male | Prevalence | 59.75 (15.73%) | 90.26 (23.76%) | 8.67 (2.28%) | -39.18 (-10.32%) |
| Tonga | Male | DALYs | 1.15 (8.88%) | 2.73 (21.09%) | 0.29 (2.21%) | -1.87 (-14.43%) |
| Tonga | Female | Prevalence | 117.22 (28.16%) | 112.88 (27.12%) | 47.88 (11.5%) | -43.54 (-10.46%) |
| Tonga | Female | DALYs | 2.92 (21.01%) | 3.37 (24.3%) | 1.55 (11.17%) | -2.01 (-14.46%) |
| Vanuatu | Male | Prevalence | 680.33 (128.5%) | 113.9 (21.51%) | 612.76 (115.74%) | -46.34 (-8.75%) |
| Vanuatu | Male | DALYs | 21.29 (123.61%) | 3.46 (20.09%) | 19.71 (114.47%) | -1.89 (-10.95%) |
| Vanuatu | Female | Prevalence | 850.38 (173.94%) | 215.18 (44.02%) | 659.91 (134.98%) | -24.72 (-5.06%) |
| Vanuatu | Female | DALYs | 26.31 (164.98%) | 6.37 (39.91%) | 21.14 (132.56%) | -1.2 (-7.5%) |
| Singapore | Male | Prevalence | 14277.13 (94.81%) | 1124.41 (7.47%) | 13686.09 (90.89%) | -533.37 (-3.54%) |
| Singapore | Male | DALYs | 429.3 (83.6%) | 1.71 (0.33%) | 452.78 (88.17%) | -25.19 (-4.91%) |
| Singapore | Female | Prevalence | 19894.13 (113.03%) | 4961.92 (28.19%) | 16011.55 (90.97%) | -1079.34 (-6.13%) |
| Singapore | Female | DALYs | 602.69 (101.48%) | 117.65 (19.81%) | 524.41 (88.3%) | -39.38 (-6.63%) |
| Viet Nam | Male | Prevalence | 232997.28 (95.79%) | 111192.25 (45.71%) | 151148.29 (62.14%) | -29343.26 (-12.06%) |
| Viet Nam | Male | DALYs | 8680.3 (76.04%) | 5176.56 (45.34%) | 6750.65 (59.13%) | -3246.9 (-28.44%) |
| Viet Nam | Female | Prevalence | 263601.75 (90.73%) | 129535.59 (44.59%) | 156104.84 (53.73%) | -22038.68 (-7.59%) |
| Viet Nam | Female | DALYs | 9488.35 (69.52%) | 6186.89 (45.33%) | 6942.6 (50.87%) | -3641.14 (-26.68%) |
| Micronesia (Federated States of) | Male | Prevalence | 127.59 (29.06%) | 163.83 (37.32%) | -3.91 (-0.89%) | -32.33 (-7.36%) |
| Micronesia (Federated States of) | Male | DALYs | 3.26 (22.64%) | 4.9 (34.01%) | -0.13 (-0.87%) | -1.51 (-10.5%) |
| Micronesia (Federated States of) | Female | Prevalence | 166.89 (36.39%) | 192.78 (42.04%) | 3.23 (0.7%) | -29.12 (-6.35%) |
| Micronesia (Federated States of) | Female | DALYs | 4.46 (30%) | 5.71 (38.4%) | 0.1 (0.69%) | -1.35 (-9.09%) |
| China | Male | Prevalence | 5262938.95 (63.03%) | 5777144.85 (69.19%) | 2079540.22 (24.91%) | -2593746.12 (-31.06%) |
| China | Male | DALYs | 214827.19 (52.43%) | 288591.51 (70.43%) | 99121.25 (24.19%) | -172885.57 (-42.19%) |
| China | Female | Prevalence | 7408989.56 (74.57%) | 7345363.27 (73.93%) | 2749031.23 (27.67%) | -2685404.94 (-27.03%) |
| China | Female | DALYs | 315246.87 (65.02%) | 379515.72 (78.28%) | 131309.36 (27.08%) | -195578.22 (-40.34%) |
| Guam | Male | Prevalence | 523.21 (73.92%) | 500.87 (70.77%) | 81.45 (11.51%) | -59.11 (-8.35%) |
| Guam | Male | DALYs | 14.64 (62.66%) | 14.48 (61.96%) | 2.58 (11.05%) | -2.42 (-10.35%) |
| Guam | Female | Prevalence | 691.09 (103.04%) | 557.21 (83.08%) | 188.63 (28.12%) | -54.74 (-8.16%) |
| Guam | Female | DALYs | 19.81 (91.61%) | 16.07 (74.31%) | 5.87 (27.14%) | -2.13 (-9.84%) |
| Cook Islands | Male | Prevalence | 7.35 (7.06%) | 50.68 (48.63%) | -34.36 (-32.97%) | -8.97 (-8.61%) |
| Cook Islands | Male | DALYs | 0.03 (0.88%) | 1.46 (43.83%) | -1.06 (-31.86%) | -0.37 (-11.09%) |
| Cook Islands | Female | Prevalence | 27.64 (27.89%) | 58.63 (59.17%) | -22.94 (-23.15%) | -8.06 (-8.13%) |
| Cook Islands | Female | DALYs | 0.65 (20.66%) | 1.68 (53.43%) | -0.7 (-22.36%) | -0.33 (-10.4%) |
| Japan | Male | Prevalence | 89045.91 (16.99%) | 101183.79 (19.3%) | -1527.03 (-0.29%) | -10610.85 (-2.02%) |
| Japan | Male | DALYs | 1830.87 (10.49%) | 2296.9 (13.16%) | -49.31 (-0.28%) | -416.72 (-2.39%) |
| Japan | Female | Prevalence | 166623.88 (27.63%) | 172061.68 (28.53%) | 12418.85 (2.06%) | -17856.64 (-2.96%) |
| Japan | Female | DALYs | 4118.29 (21.3%) | 4293.12 (22.21%) | 387.03 (2%) | -561.86 (-2.91%) |
| Kiribati | Male | Prevalence | 258 (82.21%) | 48.1 (15.33%) | 232.04 (73.93%) | -22.15 (-7.06%) |
| Kiribati | Male | DALYs | 8.07 (78.65%) | 1.47 (14.34%) | 7.51 (73.2%) | -0.91 (-8.9%) |
| Kiribati | Female | Prevalence | 356.38 (96.93%) | 91.44 (24.87%) | 285.96 (77.78%) | -21.03 (-5.72%) |
| Kiribati | Female | DALYs | 10.82 (91.63%) | 2.71 (22.94%) | 9.06 (76.69%) | -0.94 (-8%) |
| Cambodia | Male | Prevalence | 58147.32 (83.76%) | 32053.52 (46.17%) | 51756.86 (74.56%) | -25663.06 (-36.97%) |
| Cambodia | Male | DALYs | 2211.27 (72.75%) | 1447.16 (47.61%) | 2215.21 (72.88%) | -1451.1 (-47.74%) |
| Cambodia | Female | Prevalence | 62788.79 (77.33%) | 37173.64 (45.78%) | 53972.16 (66.47%) | -28357.02 (-34.92%) |
| Cambodia | Female | DALYs | 2455.99 (68.75%) | 1766.7 (49.46%) | 2336.74 (65.41%) | -1647.45 (-46.12%) |
| Fiji | Male | Prevalence | 2591.74 (58.54%) | 1776.46 (40.12%) | 1162.04 (26.25%) | -346.77 (-7.83%) |
| Fiji | Male | DALYs | 73.22 (52.37%) | 51.13 (36.58%) | 35.9 (25.68%) | -13.82 (-9.89%) |
| Fiji | Female | Prevalence | 3276.86 (68.5%) | 2335.67 (48.83%) | 1333.67 (27.88%) | -392.47 (-8.2%) |
| Fiji | Female | DALYs | 92 (61.28%) | 66.5 (44.29%) | 40.84 (27.2%) | -15.34 (-10.22%) |
| Lao People's Democratic Republic | Male | Prevalence | 13904.65 (113.52%) | 3578.04 (29.21%) | 11269.71 (92.01%) | -943.1 (-7.7%) |
| Lao People's Democratic Republic | Male | DALYs | 445.5 (102.6%) | 121.51 (27.98%) | 389.24 (89.64%) | -65.24 (-15.03%) |
| Lao People's Democratic Republic | Female | Prevalence | 14953.32 (107.08%) | 3785.38 (27.11%) | 11459.66 (82.06%) | -291.72 (-2.09%) |
| Lao People's Democratic Republic | Female | DALYs | 463.13 (97.36%) | 122.87 (25.83%) | 381.02 (80.1%) | -40.76 (-8.57%) |
| Malaysia | Male | Prevalence | 89353.43 (102.82%) | 30599.33 (35.21%) | 88393.53 (101.71%) | -29639.43 (-34.11%) |
| Malaysia | Male | DALYs | 3072.85 (80.79%) | 1240.29 (32.61%) | 3673.5 (96.59%) | -1840.94 (-48.4%) |
| Malaysia | Female | Prevalence | 66019 (58.21%) | 32002.18 (28.22%) | 89330.02 (78.76%) | -55313.21 (-48.77%) |
| Malaysia | Female | DALYs | 2282.29 (48.82%) | 1370.53 (29.32%) | 3598.97 (76.99%) | -2687.2 (-57.48%) |
| Marshall Islands | Male | Prevalence | 35.7 (20.41%) | 67.74 (38.72%) | -18.19 (-10.4%) | -13.85 (-7.92%) |
| Marshall Islands | Male | DALYs | 0.83 (14.23%) | 2.04 (35.06%) | -0.59 (-10.11%) | -0.62 (-10.72%) |
| Marshall Islands | Female | Prevalence | 35.65 (19.94%) | 66.25 (37.05%) | -17.88 (-10%) | -12.72 (-7.11%) |
| Marshall Islands | Female | DALYs | 0.86 (14.7%) | 1.98 (33.93%) | -0.57 (-9.76%) | -0.55 (-9.46%) |
| Brunei Darussalam | Male | Prevalence | 1030.51 (81.22%) | 112.87 (8.9%) | 977.28 (77.02%) | -59.64 (-4.7%) |
| Brunei Darussalam | Male | DALYs | 32.52 (72.57%) | 1.75 (3.91%) | 33.66 (75.12%) | -2.9 (-6.46%) |
| Brunei Darussalam | Female | Prevalence | 1140.69 (95.31%) | 255.26 (21.33%) | 973.87 (81.37%) | -88.45 (-7.39%) |
| Brunei Darussalam | Female | DALYs | 35.04 (83.67%) | 5.8 (13.84%) | 33 (78.78%) | -3.75 (-8.95%) |
| Australia | Male | Prevalence | 61073.97 (86.99%) | 15976.27 (22.76%) | 44978.38 (64.07%) | 119.32 (0.17%) |
| Australia | Male | DALYs | 1838.99 (80.91%) | 392.35 (17.26%) | 1430.33 (62.93%) | 16.31 (0.72%) |
| Australia | Female | Prevalence | 76047.71 (87.56%) | 17731.04 (20.42%) | 57241 (65.91%) | 1075.67 (1.24%) |
| Australia | Female | DALYs | 2231.72 (81.26%) | 444.17 (16.17%) | 1777.87 (64.73%) | 9.68 (0.35%) |
| American Samoa | Male | Prevalence | 96.5 (43.7%) | 115.82 (52.46%) | -1.66 (-0.75%) | -17.66 (-8%) |
| American Samoa | Male | DALYs | 2.47 (33.44%) | 3.34 (45.22%) | -0.05 (-0.72%) | -0.82 (-11.06%) |
| American Samoa | Female | Prevalence | 121.72 (55.94%) | 133.79 (61.48%) | 3.44 (1.58%) | -15.51 (-7.13%) |
| American Samoa | Female | DALYs | 3.24 (45.34%) | 3.85 (53.89%) | 0.11 (1.52%) | -0.72 (-10.06%) |
| New Zealand | Male | Prevalence | 10329.44 (68.08%) | 2740.72 (18.06%) | 8064.03 (53.15%) | -475.31 (-3.13%) |
| New Zealand | Male | DALYs | 312.04 (63.06%) | 66.12 (13.36%) | 258.81 (52.3%) | -12.89 (-2.6%) |
| New Zealand | Female | Prevalence | 11893.46 (62.91%) | 2995.6 (15.84%) | 9394.98 (49.69%) | -497.12 (-2.63%) |
| New Zealand | Female | DALYs | 349.99 (58.47%) | 73.37 (12.26%) | 293.2 (48.98%) | -16.58 (-2.77%) |
| Papua New Guinea | Male | Prevalence | 44369.56 (180.32%) | 4736.73 (19.25%) | 42410.6 (172.36%) | -2777.77 (-11.29%) |
| Papua New Guinea | Male | DALYs | 1415.41 (171.21%) | 141.45 (17.11%) | 1401.22 (169.5%) | -127.26 (-15.39%) |
| Papua New Guinea | Female | Prevalence | 40157.99 (178.17%) | 3459.35 (15.35%) | 38831.74 (172.28%) | -2133.1 (-9.46%) |
| Papua New Guinea | Female | DALYs | 1267.37 (168.91%) | 103.91 (13.85%) | 1270.6 (169.34%) | -107.14 (-14.28%) |
| Mongolia | Male | Prevalence | 8585.37 (94.99%) | 2928.97 (32.41%) | 5860.28 (64.84%) | -203.88 (-2.26%) |
| Mongolia | Male | DALYs | 281.28 (90.05%) | 92.22 (29.52%) | 199.65 (63.91%) | -10.59 (-3.39%) |
| Mongolia | Female | Prevalence | 10754.62 (108.27%) | 4478.01 (45.08%) | 7064.55 (71.12%) | -787.94 (-7.93%) |
| Mongolia | Female | DALYs | 338.35 (104.57%) | 134.29 (41.5%) | 227.64 (70.35%) | -23.59 (-7.29%) |
| Samoa | Male | Prevalence | 395.19 (48.3%) | 230.79 (28.21%) | 228.14 (27.88%) | -63.74 (-7.79%) |
| Samoa | Male | DALYs | 11.17 (41.81%) | 6.68 (24.98%) | 7.28 (27.22%) | -2.78 (-10.39%) |
| Samoa | Female | Prevalence | 462.13 (56.3%) | 221.51 (26.98%) | 298.47 (36.36%) | -57.85 (-7.05%) |
| Samoa | Female | DALYs | 13.29 (50.51%) | 6.41 (24.37%) | 9.38 (35.64%) | -2.5 (-9.51%) |
| Philippines | Male | Prevalence | 359584.28 (120.32%) | 123934.95 (41.47%) | 272772.32 (91.27%) | -37122.99 (-12.42%) |
| Philippines | Male | DALYs | 12230.14 (108.87%) | 4409.82 (39.25%) | 9982.31 (88.86%) | -2161.99 (-19.25%) |
| Philippines | Female | Prevalence | 389553.17 (128.01%) | 149772.46 (49.22%) | 276698.08 (90.93%) | -36917.37 (-12.13%) |
| Philippines | Female | DALYs | 12884.29 (117.03%) | 5178.08 (47.03%) | 9764.67 (88.69%) | -2058.46 (-18.7%) |
| Nauru | Male | Prevalence | 10.38 (26.96%) | 4.03 (10.47%) | 8.61 (22.37%) | -2.27 (-5.89%) |
| Nauru | Male | DALYs | 0.3 (23.53%) | 0.12 (9.73%) | 0.28 (22.07%) | -0.11 (-8.28%) |
| Nauru | Female | Prevalence | 18.71 (48.95%) | 10.14 (26.53%) | 10.44 (27.31%) | -1.87 (-4.88%) |
| Nauru | Female | DALYs | 0.55 (43.67%) | 0.3 (24.01%) | 0.33 (26.79%) | -0.09 (-7.13%) |
| Niue | Male | Prevalence | -1.19 (-8.72%) | 4.55 (33.3%) | -4.76 (-34.81%) | -0.99 (-7.21%) |
| Niue | Male | DALYs | -0.05 (-12.44%) | 0.13 (30.79%) | -0.15 (-34.04%) | -0.04 (-9.19%) |
| Niue | Female | Prevalence | -1.94 (-10.69%) | 4.24 (23.36%) | -5.05 (-27.81%) | -1.13 (-6.24%) |
| Niue | Female | DALYs | -0.07 (-12.99%) | 0.12 (22.21%) | -0.15 (-27.43%) | -0.04 (-7.77%) |
| Republic of Korea | Male | Prevalence | 68798.15 (24.37%) | 35791.85 (12.68%) | 46397.6 (16.44%) | -13391.3 (-4.74%) |
| Republic of Korea | Male | DALYs | 1203.86 (11.9%) | 296.33 (2.93%) | 1572.57 (15.54%) | -665.04 (-6.57%) |
| Republic of Korea | Female | Prevalence | 139061.76 (41.93%) | 102405.92 (30.87%) | 60028.95 (18.1%) | -23373.1 (-7.05%) |
| Republic of Korea | Female | DALYs | 3256.68 (28.29%) | 2224.65 (19.32%) | 1969.28 (17.1%) | -937.25 (-8.14%) |
| Palau | Male | Prevalence | 67.38 (84.13%) | 50.68 (63.28%) | 23.56 (29.41%) | -6.86 (-8.56%) |
| Palau | Male | DALYs | 1.92 (75.02%) | 1.47 (57.35%) | 0.73 (28.56%) | -0.28 (-10.88%) |
| Palau | Female | Prevalence | 59 (72.29%) | 52.48 (64.31%) | 12.08 (14.81%) | -5.57 (-6.83%) |
| Palau | Female | DALYs | 1.65 (64.08%) | 1.52 (58.91%) | 0.37 (14.39%) | -0.24 (-9.21%) |
| Northern Mariana Islands | Male | Prevalence | 129.52 (60.95%) | 131.95 (62.1%) | 12.08 (5.69%) | -14.51 (-6.83%) |
| Northern Mariana Islands | Male | DALYs | 3.59 (51.08%) | 3.81 (54.24%) | 0.38 (5.47%) | -0.61 (-8.63%) |
| Northern Mariana Islands | Female | Prevalence | 162.62 (92.63%) | 161.78 (92.15%) | 12.09 (6.89%) | -11.25 (-6.41%) |
| Northern Mariana Islands | Female | DALYs | 4.58 (78.63%) | 4.66 (80.01%) | 0.38 (6.57%) | -0.46 (-7.95%) |

**Abbreviations:** CI, Confidence interval; UI, Uncertainty Interval; EAPC, Estimated Annual Percentage Change

**Notes:**Sex-stratified decomposition was performed separately for males and females using the same Shapley decomposition framework. Values outside parentheses represent the estimated absolute contribution of each component within each sex, and values in parentheses represent the corresponding contribution relative to the sex-specific burden in 1990. Component percentages sum algebraically to the sex-specific overall percentage change from baseline rather than to 100%. Positive values indicate increasing effects, whereas negative values indicate offsetting effects.

| **Appendix Table 12. ARIMA projections of age-standardised refractive disorder rates globally and by WHO region in 2030** | | | | | | | |
| --- | --- | --- | --- | --- | --- | --- | --- |
| **Location** | **Indicator** | **Measure** | **Observed 2023** | **Projected 2030** | **95% prediction interval** | **Absolute change** | **Change from 2023 (%)** |
| Global | ASPR | Prevalence | 1493.26 | 1414.72 | 1364.45–1464.99 | -78.54 | -5.26 |
| Global | ASDR | DALYs | 58.50 | 54.69 | 52.15–57.24 | -3.81 | -6.51 |
| African Region | ASPR | Prevalence | 921.38 | 823.66 | 766.06–881.26 | -97.72 | -10.61 |
| African Region | ASDR | DALYs | 39.67 | 35.40 | 33.06–37.74 | -4.27 | -10.77 |
| Eastern Mediterranean Region | ASPR | Prevalence | 1629.20 | 1620.96 | 1534.86–1707.06 | -8.24 | -0.51 |
| Eastern Mediterranean Region | ASDR | DALYs | 64.11 | 65.76 | 59.95–71.57 | 1.65 | 2.58 |
| European Region | ASPR | Prevalence | 1259.46 | 1236.76 | 1220.82–1252.70 | -22.70 | -1.80 |
| European Region | ASDR | DALYs | 41.28 | 40.56 | 40.07–41.04 | -0.72 | -1.75 |
| Region of the Americas | ASPR | Prevalence | 1493.00 | 1480.94 | 1449.72–1512.17 | -12.06 | -0.81 |
| Region of the Americas | ASDR | DALYs | 48.89 | 49.12 | 48.41–49.84 | 0.23 | 0.47 |
| South-East Asia Region | ASPR | Prevalence | 2289.63 | 1913.20 | 1693.46–2132.95 | -376.43 | -16.44 |
| South-East Asia Region | ASDR | DALYs | 91.01 | 71.32 | 61.26–81.39 | -19.69 | -21.63 |
| Western Pacific Region | ASPR | Prevalence | 1382.90 | 1317.70 | 1282.64–1352.76 | -65.20 | -4.71 |
| Western Pacific Region | ASDR | DALYs | 59.85 | 55.00 | 52.66–57.34 | -4.85 | -8.10 |

**Abbreviations:** ASPR, age-standardised prevalence rate; ASDR, age-standardised DALY rate.

**Note:** Forecasts were generated from annual age-standardised rates for 1990–2023 using region-specific optimal ARIMA models. Rates are expressed per 100,000 population. Changes are calculated relative to the observed 2023 rate.
